# In roots of *Arabidopsis thaliana*, the damage-associated molecular pattern AtPep1 is a stronger elicitor of immune signalling than flg22 or the chitin heptamer

**DOI:** 10.1371/journal.pone.0185808

**Published:** 2017-10-03

**Authors:** Lorenzo Poncini, Ines Wyrsch, Valérie Dénervaud Tendon, Thomas Vorley, Thomas Boller, Niko Geldner, Jean-Pierre Métraux, Silke Lehmann

**Affiliations:** 1 Department of Biology, University of Fribourg, Fribourg, Switzerland; 2 Department of Environmental Sciences, University of Basel, Basel, Switzerland; 3 Department of Plant Molecular Biology, University of Lausanne, Lausanne, Switzerland; Iowa State University, UNITED STATES

## Abstract

Plants interpret their immediate environment through perception of small molecules. Microbe-associated molecular patterns (MAMPs) such as flagellin and chitin are likely to be more abundant in the rhizosphere than plant-derived damage-associated molecular patterns (DAMPs). We investigated how the *Arabidopsis thaliana* root interprets MAMPs and DAMPs as danger signals. We monitored root development during exposure to increasing concentrations of the MAMPs flg22 and the chitin heptamer as well as of the DAMP AtPep1. The tissue-specific expression of defence-related genes in roots was analysed using a toolkit of promoter::YFP_N_ lines reporting jasmonic acid (JA)-, salicylic acid (SA)-, ethylene (ET)- and reactive oxygen species (ROS)- dependent signalling. Finally, marker responses were analysed during invasion by the root pathogen *Fusarium oxysporum*. The DAMP AtPep1 triggered a stronger activation of the defence markers compared to flg22 and the chitin heptamer. In contrast to the tested MAMPs, AtPep1 induced SA- and JA-signalling markers in the root and caused a severe inhibition of root growth. Fungal attack resulted in a strong activation of defence genes in tissues close to the invading fungal hyphae. The results collectively suggest that AtPep1 presents a stronger danger signal to the *Arabidopsis* root than the MAMPs flg22 and chitin heptamer.

## Introduction

Roots engage in diverse interactions with their biotic environment, some being mutually beneficial (e.g. symbioses) while others are detrimental to the plant but serve the microorganism (e.g. plant diseases) [1,2]. Besides these opposing cases, there appear to be a number of seemingly neutral associations whose exact impact on the partners has still to be identified. Following rapid advances in large-scale sequencing techniques, the microbial communities inhabiting the phyllo- and rhizosphere of plants have gained particular attention in recent years [3,4]. The root microbiome not only influences nutrient availability and thus plant development, it also affects plant immunity towards root and shoot pathogens [5].

In the rhizosphere, roots are in permanent contact with an immense number of microorganisms of variable composition, many of which are bacteria and fungi [6]. The elicitors flagellin and chitin, representing contact with bacterial and fungal microbes, are perceived by pattern recognition receptors (PRRs) of the plant. The perception of microbe-associated molecular patterns (MAMPs) leads to a series of downstream events including the generation of reactive oxygen species (ROS), the phosphorylation of mitogen-activated kinases (MAP kinases) as well as transcriptional reprogramming, culminating in pattern-triggered immunity (PTI) [7,8]. The phytohormones salicylic acid (SA), jasmonic acid (JA) and ethylene (ET) are central regulators of plant immunity and orchestrate local as well as systemic immune responses in a complex network of synergistic and antagonistic interactions [9–11].

PTI responses were first studied in leaves but have also been reported in roots [12–16]. In the natural environment of roots, flagellin and chitin are assumed to be omnipresent molecules and are also components of organisms neutral or even beneficial towards plants, raising the question if these molecules are likely to function as a general signal of danger for the root. Opposed to non-self molecules such as flagellin, a class of endogenous elicitors known as damage-associated molecular patterns (DAMPs) is generated when plant tissue is damaged [7,17]. For example, microbial enzymes can release fragments of the plant cell wall such as oligogalacturonides, which entail signalling events similar to those described after MAMP recognition and can protect *Arabidopsis* leaves against fungal infection [18].

Another class of endogenous elicitors is composed of small peptides and has been termed Peps (plant elicitor peptides) [19,20]. The first member of the Pep family, AtPep1, was isolated from *Arabidopsis* leaf tissue and consists of the 23 C-terminal amino acids of its precursor PROPEP1 [21]. The leucine-rich repeat receptor-like kinases AtPEPR1 and AtPEPR2 were demonstrated to mediate Pep-dependent responses [22,23]. The 8 *PROPEP* genes identified in *Arabidopsis* differ with respect to their tissue-specific expression patterns as well as regarding the subcellular localization of the encoded proteins [24]. *PROPEP* genes are present in many angiosperms, amongst them important crops such as maize, rice and potato, but the corresponding Peps are usually not recognized between different plant families [25]. Pep-dependent signalling increases host resistance towards bacterial or fungal pathogens and protects the plant against herbivores [26–29]. In addition to their role in plant immunity there is increasing evidence that Peps also have developmental functions in plants [24,30,31].

In the present study, we hypothesize that the presence of a DAMP such as AtPep1 is interpreted as a stronger alarm signal by the root when compared to flagellin or chitin because these MAMPs are an abundant component of the rhizosphere. We analyse early PTI responses, elicitor-dependent growth inhibition as well as transcriptional regulation of defence genes in the root following treatment with equimolar concentrations of flg22, the chitin heptamer (chi7) and AtPep1. In addition we deployed a series of promoter-reporter constructs enabling precise localization of gene activation to understand which tissues and developmental zones of the root respond to the stimulus. Defence gene responses are also analysed during infection with the fungal root pathogen *Fusarium oxysporum*.

## Materials and methods

### Plant growth conditions

Plant material used was *Arabidopsis thaliana* Col-0, the mutants *fls2* (SAIL_691C4; for root growth inhibition) [32], *fls2* (SALK_062054C; for MAPK phosphorylation) [33], *cerk1-2* (GABI_096F09) [34], *pepr1 pepr2* (SALK_059281 (*pepr1*) and SALK_098161 (*pepr2*)) [23] as well as several transgenic lines that are described below. For ROS measurements and MAP kinase phosphorylation seeds were surface-sterilized and grown on 0.5x Murashige and Skoog (MS) basal medium containing 0.5% sucrose and 0.8% agar under a 24 h-photoperiod at 20°C. For elicitor treatments of *promoter*::*YFP*_*N*_ lines seeds were surface-sterilized and grown vertically in square petri-dishes on 0.5x Murashige and Skoog (MS) basal medium containing 0.5 g/l MES and 0.8% agar at pH 5.7 at 22°C day / 18°C night temperature under 16 h of light. For root growth inhibition assays and quantitative PCR seeds were surface-sterilized and grown vertically in square petri-dishes on 0.5x Murashige and Skoog (MS) basal medium containing 0.5 g/l MES and 0.8% agar at pH 5.7 at 22°C under 12 h of light. For inoculation with *Fusarium oxysporum* seeds were surface-sterilized, suspended in 0.2% agar and placed in batches of 10 seeds per well in 12-well plates onto a foam disk floating on 3 ml of liquid 0.5x Murashige and Skoog (MS) basal medium containing 0.5 g/l MES at pH 5.7 in each well (adapted from [12]). The 12-well plates were sealed with medical tape and placed in a growth chamber under a photoperiod of 10 h of light (100–150 μmol m^-2^ s^-1^) and a day / night temperature of 24°C / 22°C at 60% relative humidity.

### Transgenic lines

The promoter regions of *WRKY11* (At4g31550; 1.7 kb), *MYB51* (At1g18570; 1.7 kb), *ACS6* (At4g11280; 2.1 kb), *AOS* (At5g42650; 2 kb), *HEL/PR4* (At3g04720; 2kb), *ZAT12* (At5g59820; 2 kb), *PER5* (At1g14550; 2 kb), *ICS1* (At1g74710; 2.1 kb) and *PR1* (At2g14610; 2.1 kb) were amplified by PCR from genomic DNA of Col-0 (primers listed in S1 Table) and cloned into the KpnI site of pGreenII229NLS3xmVenus containing the coding sequence for the YFP reporter with a nuclear localization signal [35]. The obtained constructs (referred to as *promoter*::*YFP*_*N*_) were used to transform *A*. *thaliana* ecotype Col-0 using *Agrobacterium tumefaciens* and selected with BASTA. Selection of T2 seedlings on 0.5x MS medium containing 7.5 mg/l BASTA verified that the reporter lines used for subsequent analysis carry a single T-DNA insertion. Several independent insertion lines were analysed per construct and two to three lines were chosen for further analysis in the T3 generation. Using at least two reporter lines per construct increases the biological significance of the analysis, as the activity of a promoter-reporter construct is known to be dependent on its insertion site. All reporter lines have been positively tested for their ability to respond to the respective stimuli (e.g. hormones, oxidative stress; data not shown).

### Elicitor treatments

Flg22 peptide was obtained from EZBiolab (Carmell, IN, USA) and AtPep1 was obtained from Justin Lee (Halle, DE) and EZBiolab. Chitin heptamers were obtained from Elicityl SA (Crolles, FR). All elicitors were diluted in water and used at a concentration of 100 nM for microscopic analysis of reporter lines or at 1 μM for ROS measurements and MAP kinase phosphorylation. For quantitative PCR and root growth inhibition assays we used 10 nM, 100 nM or 1 μM of the respective elicitor. For quantitative PCR, 7 day old seedlings were transferred into liquid 0.5x Murashige and Skoog (MS) containing the respective elicitor molecules for 2 h before the roots were harvested. For inhibition of root growth, seedlings were grown vertically for 12 days on medium containing the respective elicitors. Primary root length was analysed using Fiji (http://fiji.sc/Fiji). For microscopic analysis of the reporter lines after elicitor treatment, 7 day old seedlings were transferred into liquid 0.5x Murashige and Skoog (MS) containing the respective elicitor molecules at a concentration of 100 nM. Based on an initial assessment of elicitor responses at 4, 8 and 24 h of treatment (S1 Fig) we selected the following treatment times for detailed microscopic analysis: 4 h (*pACS6*::*YFP*_*N*_), 6 h (*pWRKY11*::*YFP*_*N*_, *pMYB51*::*YFP*_*N*_, *pZAT12*::*YFP*_*N*_, *pPER5*::*YFP*_*N*_) or 24 h (*pAOS*::*YFP*_*N*_, *pICS1*::*YFP*_*N*_, *pHEL*::*YFP*_*N*_, *pPR1*::*YFP*_*N*_).

### Microscopy and image processing

The *promoter*::*YFP*_*N*_ lines were analysed by confocal microscopy using a Leica SPII exciting at 488 nm and detecting at 515–590 nm (initial time course elicitor treatments) and a Leica SP5 exciting at 514 nm and detecting at 525–600 nm (detailed analysis after elicitor treatments) or 525–580 nm (inoculation with *Fusarium oxysporum*). The counterstain with propidium iodide was performed by submerging the roots in 15 mg ml^-1^ propidium iodide diluted in water for 10 min followed by detection at 600–700 nm after excitation with 543 nm using a Leica SP5. Amplification of the signal (gain) was kept constant when comparing different elicitor treatments but varied between the developmental zones analysed (S5 Fig). Per line and elicitor, six seedlings were analysed of which three were selected for microscopic analysis. Each root was analysed in four developmental zones: root tip / meristem (RC), transition zone (TZ), differentiation zone (DZ) and mature zone (MZ). The optical section chosen for all root analyses was the root center as judged by the visibility of xylem vessels and/or the maximal root diameter. The pictures of the MZ were taken at about half of the entire primary root length.

Quantification of the images of *promoter*::*YFP*_*N*_ lines after elicitor treatments was performed using Fiji. All signals below a grey value threshold of 60 were excluded from quantification to avoid autofluorescence signals. The score of an image is the sum of the mean integrated density values of all particles detected that surpass the autofluorescence threshold. The average of the scores obtained from three images of replicate roots was used to calculate fold-changes between different elicitor treatments and the control. In order to increase stringency of the analysis and select the strongest responses only average values above a significance threshold of 50’000 were used directly to calculate the fold-change. Values below this threshold were set to 50’000 to exclude weak responses and focus on the stronger fluorescent signals. Fold-change values above 1.5 were defined as induction and values below 0.67 as suppression. Quantification of the images of *promoter*::*YFP*_*N*_ lines after infection with *F*. *oxysporum* was performed using Fiji. All signals below a grey value threshold of 100 were excluded from quantification to avoid autofluorescence signals.

### Measurement of ROS

For ROS assays, roots were grown vertically on 0.5x MS agar in 12 cm x 12 cm square petri dishes. When 14 days old the roots were isolated from the shoots using a razor blade. Per sample, root systems of 2 plants were placed into a well of a Lia White 96-well plate (Greiner Bio-One) containing 0.1 ml water and kept in the dark overnight. 12 samples were prepared per condition. For ROS detection, horseradish peroxidase (Sigma-Aldrich) and luminol (Sigma-Aldrich) were added as 50 μl mix per well to reach a final concentration of 10 μg ml^-1^ and 100 μM, respectively. Luminescence was measured continuously until the background signal dropped to a baseline before the elicitors were added. Elicitors were added as 10 μl stock solutions to reach a final concentration of 1 μM in the well. Luminescence was measured for 1 hour in a MicroLumat LB96P plate reader (Berthold Technologies) with a signal integration time of 1 sec.

### MAP kinase phosphorylation

For detection of MAP kinase phosphorylation, the root systems of 12 plants grown 14 days vertically on 0.5x MS agar were isolated and placed in water overnight before adding 1 μM elicitor for 10 min. Tissue (50 mg per sample) was frozen in liquid nitrogen and ground before addition of 50 μL SDS-extraction buffer (0.35 M Tris-HCl pH 6.8, 30% glycerol, 10% SDS, 0.6 M dithiothreitol, 0.012% bromophenol blue). Total proteins were separated by electrophoresis in a 12% SDS-polyacrylamide gel and transferred to a PVDF membrane according to the manufacturer’s instructions (Bio-Rad). Protein transfer was visualized by staining with Ponceau S. Polyclonal primary antibodies against phospho-p44/42 MAPK (Cell Signaling Technologies) were used with alkaline phosphatase-conjugated anti-rabbit as secondary antibodies. Signal detection was performed using CDPstar (Roche).

### Quantitative PCR

For gene expression analysis, plants were grown for 7 days vertically on 0.5x MS agar. For elicitor treatment, 20–30 seedlings were transferred to 6-well plates containing liquid 0.5x MS medium with the indicated concentrations of elicitors. After 2 hours of treatment, the roots were separated from the shoots using a scalpel blade and snap-frozen in liquid nitrogen. The tissue was ground using a TissueLyser from Qiagen. RNA was extracted using TRIzol (Invitrogen) and 2 μg were treated with DNAse I. For cDNA synthesis, 1 μg of RNA were used with the qScript cDNA synthesis kit (Quanta Biosciences). 10 ng of cDNA were used as template for qRT- PCR using SYBR Green JumpStart Taq ReadyMix (Sigma-Aldrich) with an Mx3005P (Stratagene). Samples were run as 3 technical replicates, the experiment was performed in 3 biological repetitions. Changes in gene expression were determined after normalization to the reference gene *UBQ5* (At3g62250) using the 2^-ΔΔCt^ method [36]. All primers are listed in S1 Table.

### Inoculation with *Fusarium oxysporum*

The strains 699 and 699 GFP of *Fusarium oxysporum f*.*sp*. *conglutinans* (ATCC 58110) [37] were obtained from Antonio di Pietro (Cordoba, ES). *F*. *oxysporum* was cultivated in potato dextrose agar plates at 28°C. For production of microconidia several pieces of mycelium were transferred to sucrose sodium nitrate medium and incubated for 3–4 days at 28°C and 150 rpm. The spores were isolated by filtration through 2 layers of miracloth, pelleted at 3000 g for 5 min and resuspended in liquid 0.5x MS medium at a concentration of 1*10^5^ spores / ml. This dilution was used to inoculate 10d-old seedlings growing in 12-well plates by pipetting the spore solution into the wells to reach a final concentration of 1*10^4^ spores / ml. The infection site and the MZ of infected roots were analysed at 2 dpi using confocal microscopy.

### Accession numbers

At4g31550 (*WRKY11*), At1g18570 (*MYB51*), At4g11280 (*ACS6*), At5g42650 (*AOS*), At3g04720 (*HEL/PR4*), At5g59820 (*ZAT12*), At1g14550 (*PER5*), At1g74710 (*ICS1*), At2g14610 (*PR1*), At4g05320 (*UBQ10)*

## Results

### Early PTI-responses are conserved between leaves and roots

**Fig 1:**
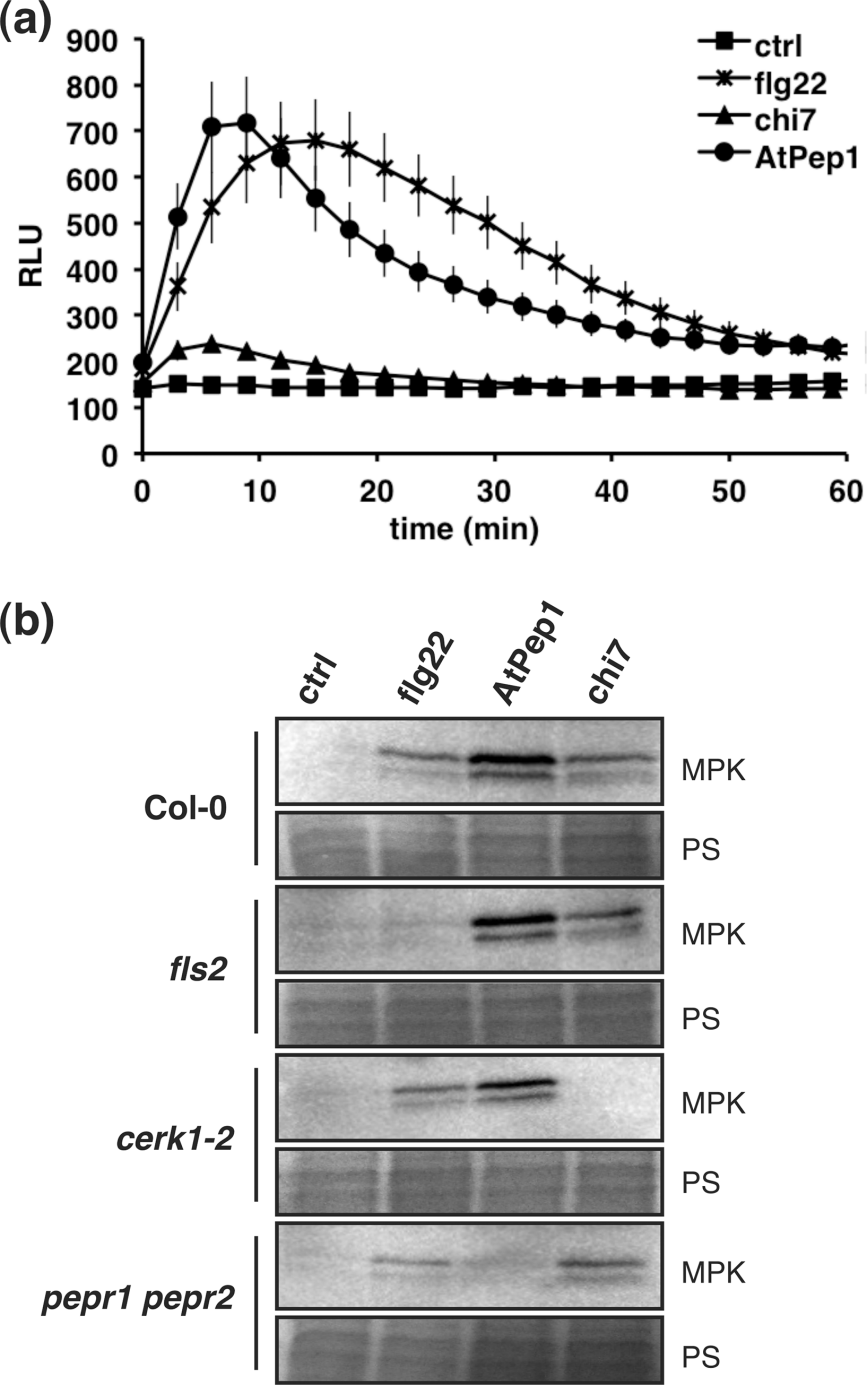
Responses of PAMP-triggered immunity in roots. (a) Luminol-based detection of ROS production in isolated roots of Col-0 seedlings treated with 1 μM flg22, chi7 or AtPep1 or without elicitor. Data represent the mean of 12 replicates ± SE. RLU—relative luminescence units (b) Activation of MAP kinases detected by western blot (MPK). Isolated roots from 2-week old seedlings of Col-0 and transgenic lines were treated with 1 μM flg22, chi7 or AtPep1 or without elicitor for 10 min before sampling. Ponceau S staining was used as loading control (PS). The experiment was performed three times with similar results.

The production of ROS is an early response of plant cells towards MAMPs such as flg22 or elf18. While this phenomenon has repeatedly been described for leaf tissue and seedlings, we compared the oxidative burst in *Arabidopsis* roots after treatment with 1 μM flg22, chi7 or AtPep1 using a luminol-based assay (Fig 1A). A significant increase in ROS accumulation was observed for all three elicitors, with flg22 and AtPep1 triggering a stronger response than chi7. The peak of luminescence consistently appeared somewhat later in treatments with flg22 compared to samples treated with chi7 and AtPep1.

Following elicitor perception, the phosphorylation of MAP kinases transduces the signal towards downstream components. This activation of MAP kinases also occurs in roots after treatment with 1 μM flg22, chi7 or AtPep1 (Fig 1B). The analysis of the respective receptor mutants *fls2*, *cerk1-2* and *pepr1 pepr2* demonstrated that also in roots, MAP kinase activation following elicitor perception depends on the previously described LRR-RLKs FLS2, CERK1, PEPR1 and PEPR2.

### AtPep1 causes a drastic inhibition of root growth

**Fig 2:**
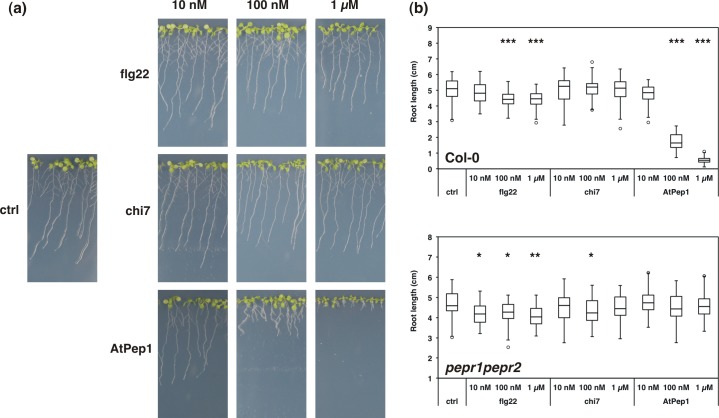
Root growth inhibition by AtPep1. (a) Col-0 wild-type and transgenic lines were germinated and grown vertically for 12 days on 0.5x MS containing 10 nM, 100 nM or 1 μM flg22, chi7 or AtPep1 or without elicitor. (b) Analysis of primary root length was performed using Fiji on images recorded under (a) and is shown as box plots representing ≥ 20 roots per sample. The experiment was performed three times for Col-0 and two times for *pepr1 pepr2*. Statistical analysis was performed using a Student’s t-test comparing with roots grown on control medium: * p < 0.05, ** p < 0.01, *** p < 0.001.

Elicitor treatment can impair plant development as was shown for flg22 by submerging seedlings in liquid medium containing flg22 [23,38]. To compare the impact of elicitors on root development we grew seedlings vertically on 0.5x MS agar plates containing 10 nM, 100 nM or 1 μM of flg22, chi7 or AtPep1. The presence of 100 nM and 1 μM AtPep1 strongly reduced the length of the primary root by a factor of 2.5 and 8.5, respectively (Fig 2). In contrast, exposure to 1 μM flg22 decreased root length only by maximally 1.5 fold across three experiments while the presence of chi7 had no reproducible effect on primary root length at either concentration. This dose-dependent effect of AtPep1 on primary root length was not observed for *pepr1 pepr2* seedlings that lack functional receptors for AtPeps. Interestingly, in a heterozygous F2 population of a *pepr1 pepr2* cross exposed to 100 nM AtPep1, plants developing the longest primary roots were mainly homozygous for the insertion in *AtPEPR2* (S2 Fig). Since the distribution of the *atpepr1* allele showed no bias towards homozygosity, the results suggest that it is mainly perception through AtPEPR2 that determines the observed root inhibition phenotype with AtPep1, similar to what has been indicated in a previous study [23].

### AtPep1 elicits a stronger induction of defence-related genes in the root than flg22 or chi7

To test the potential of flg22, chi7 and AtPep1 as danger signals to the root we generated a series of promoter-reporter constructs to monitor elicitor-driven defence signalling in specific tissues or developmental zones of the root. Expression of the YFP-derived fluorophore Venus was driven by a number of defence-related promoters (referred to hereafter as *promoter*::*YFP*_*N*_ constructs). The Venus reporter contains a nuclear localization sequence that facilitates the cell-specific detection of the fluorescent signal, allowing for the analysis of local effects in different root tissues and zones. We selected a set of defence-associated marker genes that were previously used to study root and leaf signalling and cover general immune responses such as ROS accumulation and production of antimicrobials as well as hormonal synthesis and downstream responses: *WRKY11* as an early MAMP-signalling marker [39], *MYB51* as a regulator of indole glucosinolate synthesis [40], *ACS6* reporting ET synthesis [41], *AOS* as marker for JA synthesis [42], *HEL/PR4* as downstream marker for ET/JA signalling and *ICS1* as well as *PR1* as markers for SA synthesis and signalling [43–45]. *ZAT12* and *PER5* were selected as markers associated to oxidative stress, where *PER5* encodes a peroxidase expressed following MAMP and DAMP treatment [46–48]. Based on an initial assessment of elicitor-dependent responses at 4, 8 and 24 h of treatment (S1 Fig) we selected the following treatment times for detailed microscopic analysis: 4 h (*pACS6*::*YFP*_*N*_), 6 h (*pWRKY11*::*YFP*_*N*_, *pMYB51*::*YFP*_*N*_, *pZAT12*::*YFP*_*N*_, *pPER5*::*YFP*_*N*_) or 24 h (*pAOS*::*YFP*_*N*_, *pICS1*::*YFP*_*N*_, *pHEL*::*YFP*_*N*_, *pPR1*::*YFP*_*N*_).

**Fig 3:**
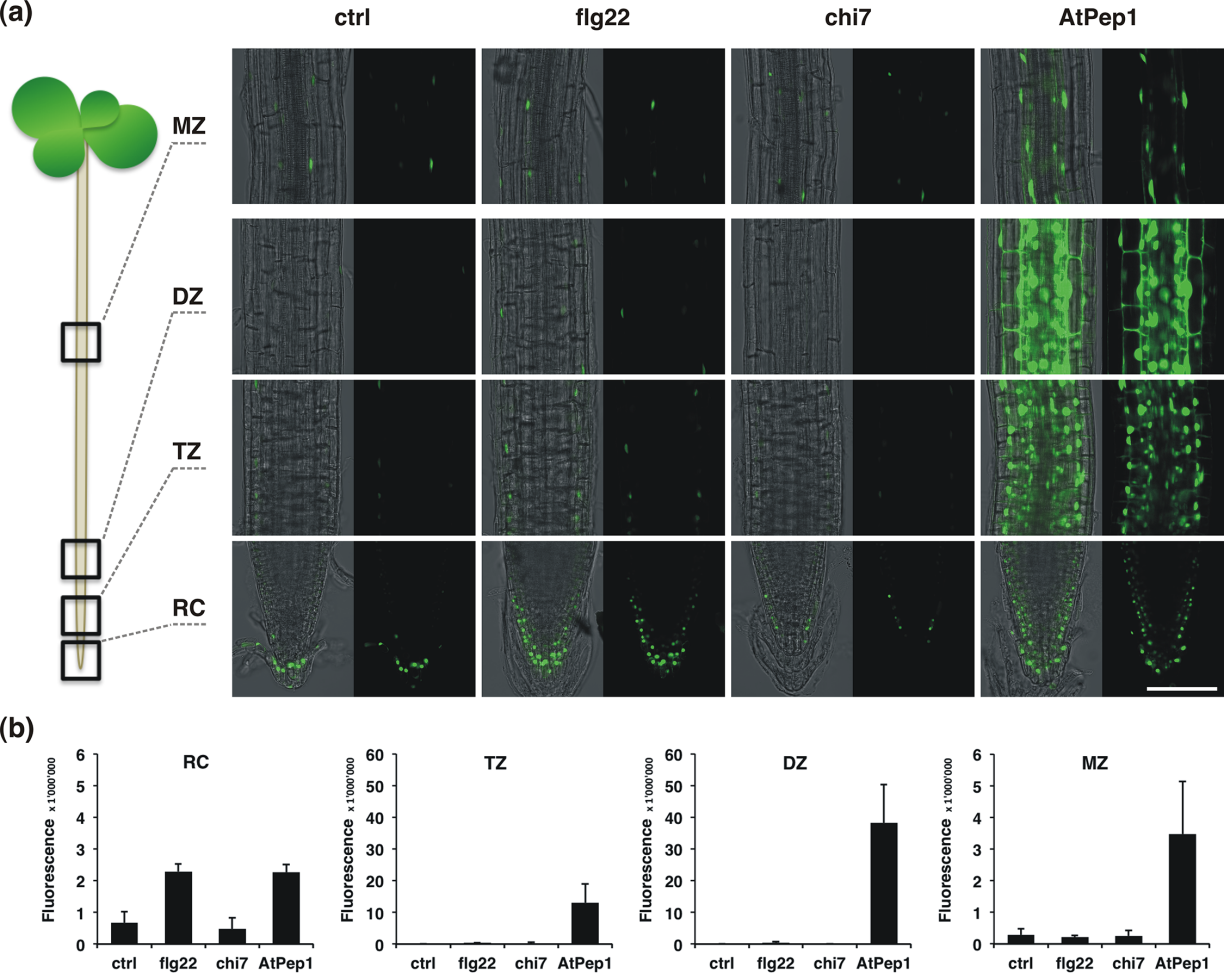
Elicitor-triggered responses of *pHEL*::*YFP*_*N*_. (a) Microscopic analysis of root developmental zones of 7-days old *pHEL*::*YFP*_*N*_ seedlings following 24 h treatment with 100 nM flg22, chi7, AtPep1 or 0.5x MS as control. RC—root cap / meristem, TZ—transition zone, DZ—differentiation zone, MZ—mature zone. Bar 100 μM. (b) Quantification of fluorescent signals from microscopic analysis of different developmental zones of *pHEL*::*YFP*_*N*_ as described under (a) using Fiji. Bars represent the mean of ≥ 3 images ± SD.

Following treatment with 100 nM of flg22, chi7, AtPep1 or 0.5x MS without elicitor, the fluorescent signal of the reporter was analysed in the root tip (RC, root cap and meristematic tissue), the transition zone (TZ), the differentiation zone (DZ, visible formation of tracheary elements and root hairs) and in the mature zone (MZ). Using confocal microscopy and subsequent signal quantification by Fiji (http://fiji.sc/Fiji), the four developmental zones were analysed in at least 3 different roots for each marker and treatment. At least two independent transformation lines were studied per construct in order to ensure biological reliability of the observed patterns, as the activity of a promoter-reporter construct is known to be dependent on its insertion site. Fig 3A gives an example of a corresponding dataset obtained using ET/JA pathway marker *pHEL*::*YFP*_*N*_. The strong AtPep1-dependent activation of *pHEL*::*YFP*_*N*_ was observed in all developmental zones analysed and was not present in roots treated with flg22, chi7 or mock solution (Fig 3 and S3E Fig). Signal quantification confirmed that the level of *pHEL*::*YFP*_*N*_ -derived fluorescence in AtPep1-treated roots clearly exceeded that in MAMP-treated roots (Fig 3B). Corresponding micrographs of the other *promoter*::*YFP*_*N*_ lines are provided in the supporting information (S3A–S3I Fig).

**Fig 4:**
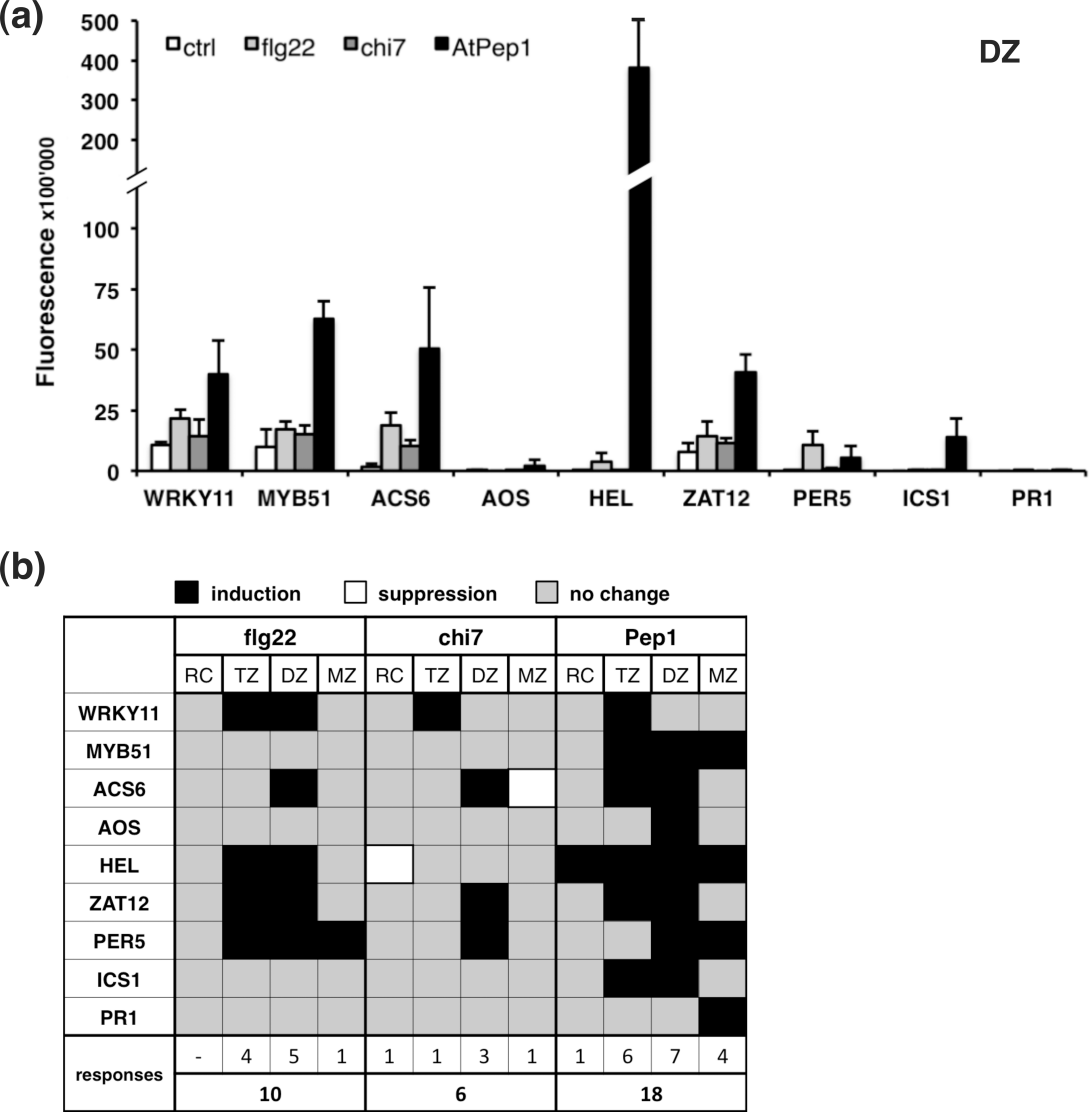
Elicitor-dependent activation of defence-related promoters in the TZ and DZ of the root. (a) Quantification of microscopic analysis of *Promoter*::*YFP*_*N*_ constructs in the differentiation zone of 7-days old seedlings following treatment with 100 nM flg22, chi7, AtPep1 or 0.5x MS as control. Images were analysed using Fiji. Bars represent the mean of ≥ 3 images ± SD. (b) Overview of inductive and suppressive responses of *Promoter*::*YFP*_*N*_ constructs in different developmental root zones analysed as described under (a). Numbers in the upper row indicate the sum of significant marker responses in one of the four analysed root zones for a given elicitor, while numbers in the lower row indicate the total sum of significant responses from all markers and all root zones for a given elicitor. Black colour indicates induction, white colour suppression and grey colour non-significant changes.

Most markers analysed showed the strongest transcriptional activation in the transition and differentiation zone. Fig 4A summarizes the YFP-derived signal intensities for all tested reporter constructs in the differentiation zone (DZ) of the root, where the majority of the markers showed a higher signal following treatment with AtPep1 when compared to flg22 or chi7. Signal quantification was also used to calculate factors of induction or repression, which are illustrated in a colour-coded table (Fig 4B). Marker responses were only considered significant if they passed a fold-change threshold of 1.5 for inductions or 0.67 for suppressions in at least two independent transformation lines for each construct, thereby focusing on the strongest responses revealing the largest expression changes (Fig 4B, S5A Fig).

The summary of significant marker changes shows that all developmental zones are able to translate elicitor signalling into transcriptional changes, although the strongest inductions of defence-associated promoters by elicitors were observed in the TZ and DZ (Fig 4A and 4B, S3A–S3I and S4 Figs). Accordingly, the sum of significant marker responses for each developmental zone is highest for the DZ (15), followed by the TZ (11), the MZ (6) and the RC (2) (Fig 4B). Although most responses observed were inductions, chi7 treatment notably resulted in repression of *pACS6*::*YFP*_*N*_ in the MZ and of *pHEL*::*YFP*_*N*_ in the RC (Fig 4B, S3C and S3E Fig, S4 Fig).

While all tested constructs responded to AtPep1 treatment with transcriptional changes in at least one developmental zone, only five out of nine markers significantly changed expression following treatment with flg22 or chi7 (Fig 4B). The number of significant marker responses for a given elicitor counted individually for the four analysed root zones was highest for the DAMP AtPep1 (n = 18), followed by flg22 responses (n = 10) and chi7 responses (n = 6) (Fig 4B). Therefore our study evidences that the DAMP AtPep1 leads to a stronger response of the selected defence genes in roots compared with the MAMPs flg22 and chi7.

### Tissue specificity of elicitor-triggered responses

The potential of the *promoter*::*YFP*_*N*_ reporter system to resolve regulatory questions at the tissue level is greatly demonstrated by the following examples. The basal expression pattern of a construct often varied depending on the developmental zone. If expression was observed in the root tip, it was mostly restricted to the RC layers (e.g. *pWRKY11*::*YFP*_*N*_ and *pHEL*::*YFP*_*N*_; Fig 3, S3A and S3E Fig). Basal expression of the marker constructs in the TZ was visible in the epidermal layer only and sometimes appeared after elicitor treatments in the central tissues beginning to differentiate (e.g. *pMYB51*::*YFP*_*N*_ and *pZAT12*::*YFP*_*N*_ after treatment with AtPep1, S3B and S3F Fig).

Inductions were mainly observed in tissues that express the respective gene at a basal level under control conditions, such as *pWRKY11*::*YFP*_*N*_, *pMYB51*::*YFP*_*N*_, and *pZAT12*::*YFP*_*N*_ throughout most root tissues of the DZ, *pACS6*::*YFP*_*N*_ in the stele or *pPER5*::*YFP*_*N*_ in the epidermis (S3 Fig). A more unusual regulation was revealed for *pHEL*::*YFP*_*N*_, which was strongly induced by AtPep1 in the TZ and particularly the DZ, where expression of this construct is low under control conditions (S3E Fig). However, in the MZ *pHEL*::*YFP*_*N*_ expression was mainly restricted to the endodermis independently of the elicitor used and varied in expression level rather than tissue localization.

AtPep1 treatment appeared to induce some constructs, e.g. *pMYB51*::*YFP*_*N*_ and *pZAT12*::*YFP*_*N*_, more strongly in the endodermis and the stele when compared to cortex and epidermis (S3B and S3F Fig). A more detailed analysis could clarify if that observation is due to the fact that stele cells are smaller than epidermal and cortical cells and therefore display more nuclear signals in a given area or if the endodermis and the vascular tissues indeed respond more strongly to AtPep1.

### AtPep1 induces JA- and SA-signalling in the root

The elicitor-triggered responses of the *promoter*::*YFP*_*N*_ lines revealed that several promoters are activated by more than one elicitor molecule (Fig 4). The early MAMP-signalling marker *pWRKY11*::*YFP*_*N*_ proved responsive to all three tested molecules. Similarly, the ROS-related constructs *pZAT12*::*YFP*_*N*_ and *pPER5*::*YFP*_*N*_ were activated by flg22, chi7 and AtPep1, even if the intensity of the response varied clearly between the different developmental zones and elicitors (Fig 4B, S4 and S5 Figs).

An interesting differential regulation was observed for the markers associated to ET signalling, *pACS6*::*YFP*_*N*_ and *pHEL*::*YFP*_*N*_. All three elicitors activated *pACS6*::*YFP*_*N*_ in the DZ, suggesting ET synthesis in the stele upon elicitor perception (Fig 4B, S3C Fig). A downstream component of ET-signalling, *pHEL*::*YFP*_*N*_, was activated by flg22 in the TZ and by AtPep1 in all root zones analysed. However, the ET or JA synthesis markers used in this study (*pACS6*::*YFP*_*N*_ and *pAOS*::*YFP*_*N*_) do not mirror the AtPep1-dependent induction of *pHEL*::*YFP*_*N*_ in all tested root zones, indicating that AtPep1 triggers an additional positive regulator of *HEL* that has yet to be identified. The *ACS* family comprises 9 genes in *Arabidopsis*, suggesting that an isoform of ACS6 might contribute to the strong upregulation of *pHEL*::*YFP*_*N*_ [49].

Notably, markers of the defence-related hormones JA and SA were activated when treated with the DAMP AtPep1 while no significant changes were observed following perception of the MAMPs flg22 and chi7. The JA-synthesis marker *pAOS*::*YFP*_*N*_ responded in the DZ, while the SA-related markers were induced in TZ and DZ (*pICS1*::*YFP*_*N*_) and the MZ (*pPR1*::*YFP*_*N*_), respectively (Fig 4B; S3D, S3H and S3I Fig). The concurrent induction of pathways for SA and JA signalling supports a function of the DAMP AtPep1 as a danger signal to the plant.

**Fig 5:**
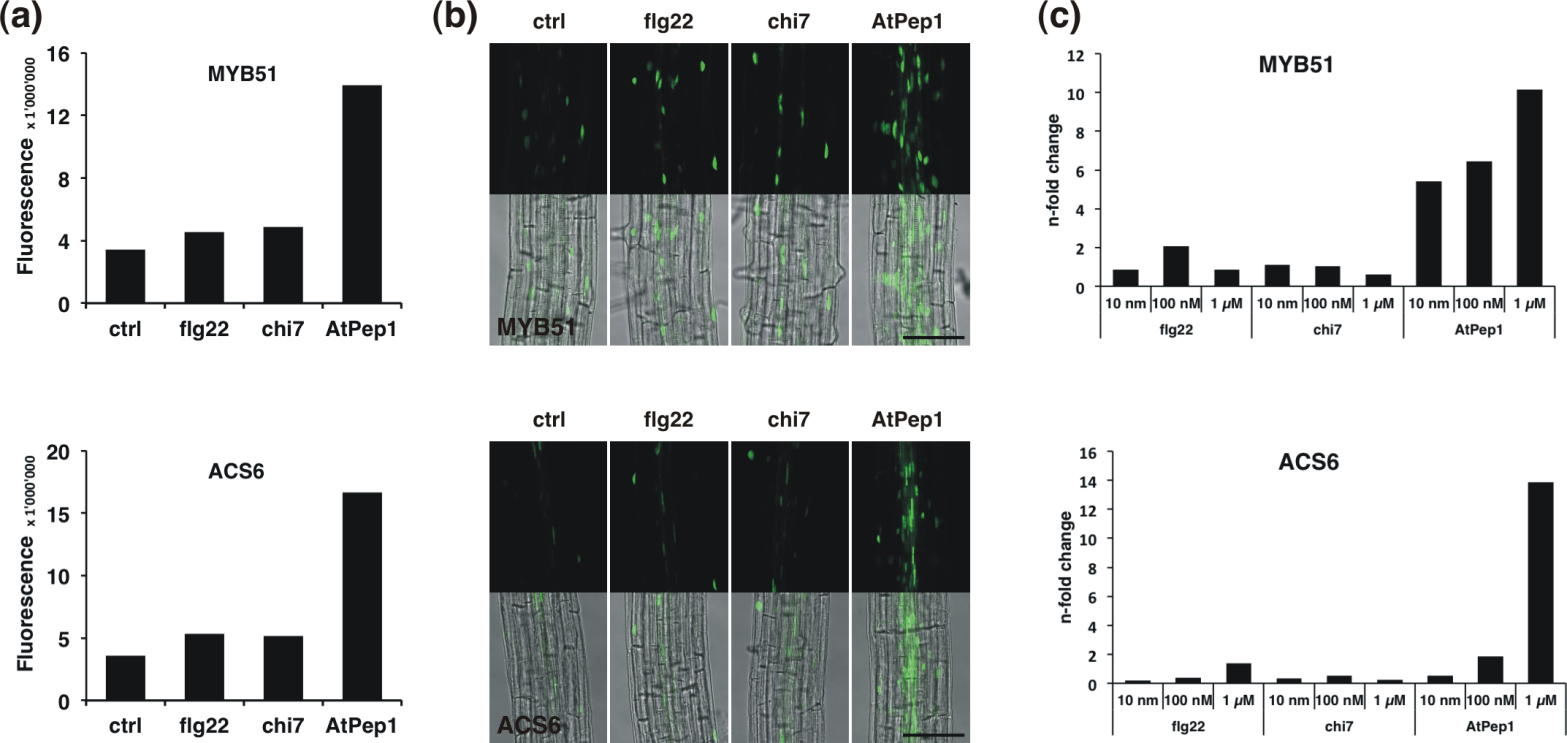
Verification of elicitor-triggered responses of *promoter*::*YFP*_*N*_ markers by qPCR. (a) Signal quantification using micrographs from roots expressing *pMYB51*::*YFP*_*N*_ and *pACS6*::*YFP*_*N*_. 7-day old seedlings were treated with 100 nM flg22, chi7 or AtPep1 or 0.5x MS without elicitor and analysed in four developmental zones. Bars represent the sum of average signals obtained for each of the four developmental zones from 2–3 independent transformation lines per construct. (b) Expression of *pMYB51*::*YFP*_*N*_ and *pACS6*::*YFP*_*N*_ in the differentiation zone of 7-day old seedlings treated with 100 nM flg22, chi7 or AtPep1 or 0.5x MS without elicitor. Bar 100 μm. (c) Expression of *MYB51* and *ACS6* analysed by quantitative RT-PCR following 2 h treatment of 7-day old seedlings with 100 nM flg22, chi7 or AtPep1 or 0.5x MS without elicitor. Transcript levels were normalized to the reference gene *UBIQUITIN5* before calculation of expression relative to the control. The experiment was repeated three times with similar results.

To confirm reliability of the *promoter*::*YFP*_*N*_ constructs we also analysed gene expression by quantitative RT-PCR (qPCR) (Fig 5). In accordance with the microscopic analysis of *pMYB51*::*YFP*_*N*_ qPCR detected the highest transcript levels of *MYB51* following treatment with AtPep1. Similarly, increased accumulation of *ACS6* mRNA induced by AtPep1 corresponded to the increased promoter activity visualized through *pACS6*::*YFP*_*N*_ and exceeded that observed after flg22 treatment (Fig 5). The analysis also clearly demonstrates that AtPep1 induces expression of *MYB51* and *ACS6* in a dose-dependent manner.

### Fungal invasion mounts a local burst of defences in the root

**Fig 6:**
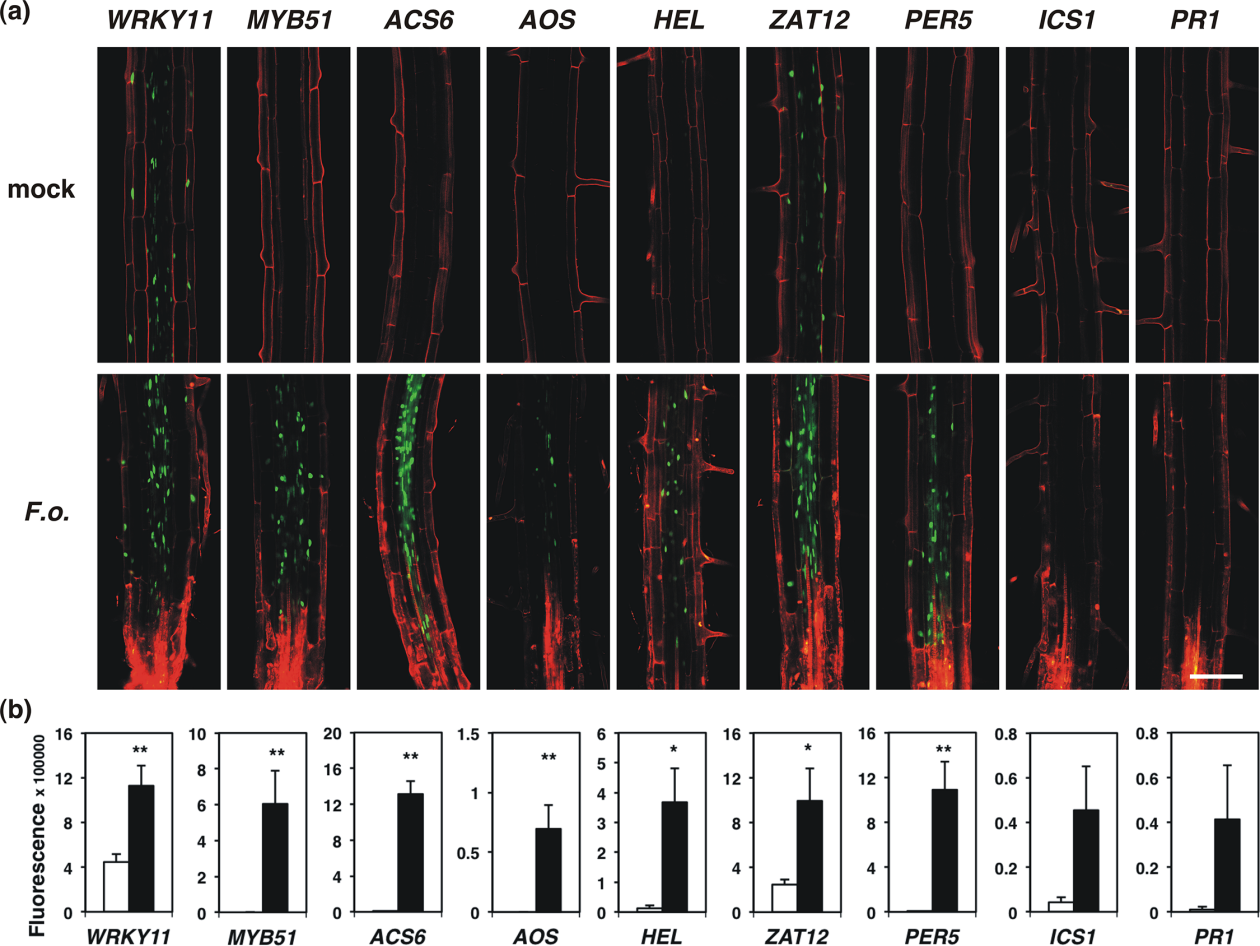
Expression of *promoter*::*YFP*_*N*_ constructs in roots infected with *F*. *oxysporum*. (a) Microscopic analysis of the infection site of 12-day old roots 2 days after inoculation with spores from *F*. *oxysporum*. Fluorescence derived from *promoter*::*YFP*_*N*_ constructs is localized in the root nuclei and shown in green, while propidium iodide was used as a counterstain of cell walls and dead cells and is shown in red. Bar 100 μm. (b) Quantification of microscopic analysis of *promoter*::*YFP*_*N*_ constructs as depicted in (a) using Fiji. Bars represent the mean of ≥ 6 images ± SE. Statistical analysis was performed using a Student’s t-test: * p < 0.05, ** p < 0.01, *** p < 0.001.

In order to see how the contact with elicitors differs from the complex situation of a real pathogen attack, we analysed the set of *promoter-YFP*_*N*_ lines during infection with the fungus *Fusarium oxysporum*. This root-invading pathogen infects a wide range of host plants and enters the root at the tip before growing towards the hypocotyl [50,51]. Seedlings were grown in 12-well plates containing liquid 0.5x MS medium and inoculated with *F*. *oxysporum* spores when 10 d old. The roots were analysed 2 days after inoculation (2 dpi) when the fungus had penetrated the root and the front of growing hyphae localized to a region around 1–5 mm distance from the tip. The analysis focused on local responses at the infection site (in close vicinity of the invading hyphae) as well as on distant effects in the MZ of the root (about 2–3 cm distance from the tip). We found that the majority of constructs tested were induced locally during *F*. *oxysporum* infection (Fig 6). Quantification of the response demonstrated that the *YFP*_*N*_-constructs with *pWRKY11*, *pMYB51*, *pZAT12*, *pACS6*, *pAOS* and *pHEL* were significantly activated at the fungal infection site (Fig 6B, S6 Fig). The markers used to report SA responses, *pICS1*::*YFP*_*N*_ and *pPR1*::*YFP*_*N*_, did not show a consistent expression pattern during fungal invasion (Fig 6, S6 Fig). Expression of ROS marker *pPER5*::*YFP*_*N*_ in infected roots exceeded that in mock-treated roots, with the response being significant in 2 out of 4 analyses (Fig 6A, S6 Fig). Using a GFP-expressing *F*. *oxysporum* strain we confirmed that marker activation occurs adjacent to the invading fungal hyphae (S7 Fig).

During these early stages of infection the responses were observed close to the hyphal front, mostly independent of the exact distance from the root tip. In contrast to this general activation of defence signalling on a local level we observed only few changes in the mature part of infected roots (S8 Fig). Thus, roots appear to respond to the local invasion of a pathogen with local activation of defence-associated genes across all tissue layers yet do not initiate a clear systemic root response at this early time point.

## Discussion

In their natural environment, roots are faced with a complex mixture of molecular signals that is likely to become locally enriched in DAMPs when the root is under attack. Early and late responses to microbial molecular patterns have been studied under a variety of experimental conditions [7]. Some of the described changes, such as callose deposition, can vary significantly dependent on the growth conditions applied, highlighting the flexibility and adaptive character of PTI signalling in nature [52]. Despite similarities in early cellular responses to MAMPs and DAMPs such as ROS accumulation, the DAMP AtPep1 causes a severe inhibition of primary root growth when continuously applied (Figs 1 and 2), pointing towards unique features of PEPR-dependent signalling when compared to flg22-mediated root responses.

The microscopic analysis of different developmental zones of elicitor-treated roots showed that AtPep1 triggered a higher number and intensity of immune marker responses when compared to flg22 or chi7 (Figs 3 and 4). The striking observation that *pHEL*::*YFP*_*N*_ is activated by AtPep1 in all tested root zones supports the idea that the DAMP presents a signal of threat to the root. *HEL* encodes a homolog of the pathogenesis-related protein PR-4 from tobacco [53] and is inducible by JA and ET treatment [45,54]. Accordingly, we found increased expression of *pAOS*::*YFP*_*N*_ and *pACS6*::*YFP*_*N*_ after AtPep1 treatment, suggesting intense JA/ET-signalling (Fig 4). Similar to the distinction between MAMPs and DAMPs observed in the present study, AtPep1 also evoked a stronger induction of the defensin gene *PDF1*.*2* in whole seedlings when compared to flg22 or elf18 [55].

Another finding that singularizes AtPep1 is its potential to co-activate markers of the SA and JA pathways (Fig 4B; S3D and S3H Fig). Neither the marker for SA-synthesis, *pICS1*::*YFP*_*N*_, nor that for JA-synthesis, *pAOS*::*YFP*_*N*_, were responding to 100 nM flg22 or chi7, while both constructs were induced by AtPep1 in the root. SA- and JA-dependent hormone signalling have repeatedly been demonstrated to work antagonistically during plant immune responses [56,57]. However, concomitant activation of SA and JA pathways has been found in several instances as well [58,59]. In particular, parallel activation of SA and JA-pathways was reported after AtPep1-treatment of leaves, when increased transcript levels of the corresponding marker genes *PR1* and *PDF1*.*2* were observed [60]. Likewise, co-activation of subsets of SA- and JA-inducible genes was detected in a transcriptome analysis of AtPep2-treated seedlings [55].

Our results demonstrate that the differentiation zone responds more often to elicitor treatment than the other developmental zones (Fig 4B). While the mature epidermal cell walls exposed to the rhizosphere have undergone impregnation with phenolic compounds, epidermal cell walls of the young, differentiating zones are less solidified and act as the site of root exudation [61]. In line with a role of the DZ in the uptake and release of compounds is the concept that a less rigid outer barrier might be accompanied by an increased perceptiveness and/or responsiveness towards potential invaders. The ET marker *pACS6*::*YFP*_*N*_ was activated in vascular tissues of the DZ following treatment with flg22 (S3C Fig). While it is conceivable that the elicitor molecules diffuse through the apoplast of epidermal and cortical cells, it appears less likely that they can pass the endodermis with the casparian strip that presents a natural diffusion barrier [62]. However, flg22 perception might trigger the production of ET, which could enter the stele and induce *pACS6*::*YFP*_*N*_. Alternatively, flg22 could be perceived directly in the vascular tissue layers if the peptide is entering the root before the diffusion barriers in the endodermis are fully formed. In this scenario, the first cells that perceive flg22 would be the *FLS2*-expressing cells in the DZ, much as was observed with our *promoter*::*YFP*_*N*_ lines and with a *pFLS2*::GUS construct by [63]. According to publicly available transcriptome analyses the transcript abundance of *FLS2* in the root appears to be generally lower than that of *PEPR1*, *PEPR2* and *CERK1* (S9 Fig, [48]). The receptors for AtPeps, *PEPR1* and *PEPR2*, are expressed throughout the entire root length, but the *PEPR2* promoter is mainly active in the stele [24,64]. In light of the observation that PEPR2 is largely responsible for AtPep1-dependent root growth inhibition (S2 Fig, [23]), this result indicates that AtPep1 can indeed pass the peripheral root tissues to bind the PEPR2 receptor in the stele. A recent study used fluorescently labelled AtPep1 to track the intracellular route of the receptor-ligand complex with PEPR1, enabling new approaches to analyse the *in vivo* diffusion properties of peptides in the root [64]. Future studies will also provide additional insight into the biochemical stability of elicitors that would affect the signalling potential of each molecule [65].

Our analysis of the *promoter*::*YFP*_*N*_ markers during root invasion by the pathogen *Fusarium oxysporum* offers a first insight into tissue-specific root immune signalling during pathogen attack and demonstrates the potential to study root-microbe interaction close to real-time. The majority of analysed markers was induced in the root zone located adjacent to the front of the progressing hyphae, illustrating the concomitant activation of several lines of defence within close proximity to the pathogen (Fig 6). Transcriptional responses that are restricted to a small number of cells are difficult to detect using quantitative PCR of whole roots, underlining the analytical power of the marker toolset generated in this study.

Using the oxidative stress marker *pZAT12*::*YFP*_*N*_ we confirmed a previous analysis based on staining with specific dyes showing that *F*. *oxysporum* causes elevated levels of ROS and nitric oxide in *Arabidopsis* roots [66]. Interestingly, *F*. *oxysporum* also appears to secrete small peptides mimicking plant alkalinizing proteins in order to facilitate disease development, thereby targeting early events in host immune signalling [67].

The increased expression of the JA synthesis marker *pAOS*::*YFP*_*N*_ during root invasion by *F*. *oxysporum* (Fig 6) could be related to the finding that JA signalling is associated with increased susceptibility to this pathogen [68]. The JA receptor complex mutant *coi1* exhibits increased resistance towards *F*. *oxysporum*, which is not a consequence of reduced JA-SA antagonism [68]. Nevertheless it has been found that SA responses decrease the susceptibility to *F*. *oxysporum* [69,70]. The lack of activation of *ICS1* and *PR1* detected in our study indicates that the selected markers are not involved in the SA-associated immune response towards *F*. *oxysporum* or that the pathogen can suppress the induction of these genes. In support of this idea, a transcriptome analysis of *F*. *oxysporum*-inoculated roots detected a decrease in *PR1* expression level [71].

Pathogen assays with leaves showed that *pepr1 pepr2* plants exhibit increased susceptibility to *P*. *syringae*, *Botrytis cinerea* and *Colletotrichum higginsianum*, suggesting that PEPR-mediated signalling is involved in immunity towards pathogens with lifestyles ranging from hemibiotrophic to necrotrophic [28,72–74]. Future studies will show how the PEPR pathway affects basal immunity to root pathogens and to which extent the defence responses described here reflect gene regulation in roots growing in natural soils.

## Conclusions

Taken together, our findings show that the DAMP AtPep1 is perceived as a stronger danger signal by the *Arabidopsis* root than the MAMPs flg22 and chi7. We therefore suggest PEPR signalling as a major component of the root surveillance apparatus involved in the interpretation of its biotic environment.

## Supporting information

S1 Table Primer sequences. (PDF)

S1 Fig Time dependency of elicitor effects on *promoter*::*YFP*_*N*_ expression in the root. Roots were analysed following treatment with 100 nM flg22, chi7, AtPep1 or 0.5x MS as control. Scale bar 200 μm.
(PDF)

S2 Fig AtPEPR2 determines root growth inhibition by AtPep1. Allele distribution of *pepr1* and *pepr2* in a segregating F2 population of a *pepr1 pepr2* cross with *promoter*::*YFP*_*N*_ lines selected for loss of root inhibition on 0.5x MS containing 100 nM AtPep1. Numbers represent individual plants genotyped for the respective *pepr* allele.
(PDF)

S3 Fig Effects of elicitors on the expression of *promoter*::*YFP*_*N*_ in the root. Roots were analysed following treatment with 100 nM flg22, chi7, AtPep1 or 0.5x MS as control. Scale bar 100 μm. Signal amplification might differ between developmental zones (S5 Fig).
(PDF)

S4 Fig Quantification of elicitor-triggered responses of defence-associated promoters in the root. Quantification of microscopic analysis of *promoter*::*YFP*_*N*_ constructs in two independent lines of 7-days old seedlings following treatment with 100 nM flg22, chi7, AtPep1 or 0.5x MS as control. Images were analysed using Fiji. Bars represent the mean of ≥ 3 images ± SD. An asterisk indicates a mean of two values.
(PDF)

S5 Fig Summary of elicitor-triggered responses and amplification settings used for microscopic analysis. (a) Overview of inductive and suppressive responses of *promoter*::*YFP*_*N*_ constructs expressed in 7-days old seedlings following treatment with 100 nM flg22, chi7, AtPep1 or 0.5x MS as control. Numbers represent the factors calculated as fold-change for 2 independent transformation lines per construct. A third line was analysed for *ACS6*::*YFP*_*N*_ and *HEL*::*YFP*_*N*_ constructs to confirm whether or not chi7 can cause a suppression of these markers. Black colour of a field indicates induction, white colour suppression and grey colour non-significant changes. (b) Table summarizing the amplification settings (gain) used for confocal microscopic analysis of *promoter*::*YFP*_*N*_ constructs listed as an indicator for the expression level of the respective construct.
(PDF)

S6 Fig Quantification of *promoter*::*YFP*_*N*_ constructs at the infection site of roots invaded by *F*. *oxysporum*. (a) Quantification of microscopic analysis of *F*. *oxysporum—*infected roots expressing *promoter*::*YFP*_*N*_ constructs in independent transformation lines when compared to Fig 6. Bars represent the mean of ≥ 6 images ± SE. (b) Quantification of microscopic analysis of *pHEL*::*YFP*_*N*_, *pPER5*::*YFP*_*N*_ and *pICS1*::*YFP*_*N*_ constructs using Fiji. Bars represent the mean of ≥ 20 images ± SE. Statistical analysis was performed using a Student’s t-test: * p < 0.05, ** p < 0.01, *** p < 0.001. (b.t.) indicates that all signals were below the autofluorescence threshold.
(PDF)

S7 Fig Induction of *promoter*::*YFP*_*N*_ constructs adjacent to invading hyphae of GFP-labelled *F*. *oxysporum*. Microscopic analysis of the responses of 12-day old roots in the mature part 2 days after inoculation with spores from F. oxysporum. Fluorescence derived from promoter::YFP_N_ constructs is localized in the root nuclei while fungal hyphae are green filamentous structures. Bar 100 μm.
(PDF)

S8 Fig Expression of *promoter*::*YFP*_*N*_ constructs in the mature part of roots infected with *F*. *oxysporum*. (a) Microscopic analysis of the responses of 12-day old roots in the mature part 2 days after inoculation with spores from *F*. *oxysporum*. Fluorescence derived from *promoter*::*YFP*_*N*_ constructs is localized in the root nuclei and shown in green, while propidium iodide was used as a counterstain of cell walls and dead cells and is shown in red. Bar 100 μm. (b) Quantification of microscopic analysis of *promoter*::*YFP*_*N*_ constructs as depicted in (A) using Fiji. Bars represent the mean of ≥ 6 images ± SE. (C) Quantification of microscopic analysis of *F*. *oxysporum—*infected roots expressing *promoter*::*YFP*_*N*_ constructs in independent transformation lines when compared to Fig 6A and 6B. Bars represent the mean of ≥ 6 images ± SE. Statistical analysis was performed using a Student’s t-test: * p < 0.05, ** p < 0.01. (b.t.) indicates that all signals were below the autofluorescence threshold.
(PDF)

S9 Fig Expression of MAMP/DAMP receptor genes in roots. (PDF)
