# Supplementary material for: In roots of Arabidopsis thaliana, the damage-associated molecular pattern AtPep1 is a stronger elicitor of immune signalling than flg22 or the chitin heptamer
Source: PLoS One. 2017 Oct 3;12(10):e0185808. doi: 10.1371/journal.pone.0185808 (PMC5626561; doi:10.1371/journal.pone.0185808)
Supplement: S1 Table — (PDF) [file pone.0185808.s001.pdf]

S1 Table: Primer sequences.

Promoter amplification for promoter::YFP_N constructs

pWRKY11::YFP_N	fw 5' ATTGGGTACCCTTCCCCACCCATATATAGCCA 3'<br>rv 5' GCCCGGTACCGATGATTTCTTGGTCTGAGGAT 3'
pMYB51::YFP_N	fw 5' ATTGGGTACCTGTACTAAAGAACTACTGTAA 3'<br>rv 5' GCCCGGTACCTCTTGATTCTTCAAACTTAGCT 3'
pACS6::YFP_N	fw 5' ATTTGGTACCATGTCAACTAAAACCG 3'<br>rv 5' AAGCGGTACCTTTTTGTTTCTTCTTTA 3'
pAOS::YFP_N	fw 5' ATTGGGTACCGAAAACTGGTAAGCTT 3'<br>rv 5' GCCCGGTACCCTATTCGAAACAGTGG 3'
pHEL::YFP_N	fw 5' TAAGGGTACCTAAATGACATGAGATG 3'<br>rv 5' TGATGGTACCGATCGATAAGTCTTTG 3'
pZAT12::YFP_N	fw 5' GTGTGGTACCTTTTAGGCTCGTAAAG 3'<br>rv 5' TCGCGGTACCTTTTCTTCTGATGATG 3'
pPER5::YFP_N	fw 5' AATGGTACCCTCAGTGCGTAGTAGTGAGT 3'<br>rv 5' AATGGTACCATTTGTAGATCTCACTTGGTATA 3'
pICS1::YFP_N	fw 5' CTTAGGTACCCAGACTCCAGAAGACGAC 3'<br>rv 5' GTGTGGTACCTGCAGAAATTCGTAAAGTG 3'
pPR1::YFP_N	fw 5' ATTAGGTACCAAGTCATTTACAAAG 3'<br>rv 5' TAACGGTACCTTTTCTAAGTTGATAATG 3'

Analysis of gene expression by qPCR

UBIQ10	fw 5' GGCCTTGTATAATCCCTGATGAATAAG 3'<br>rv 5' AAAGAGATAACAGGAACGGAAACATAG 3'
PER5	fw 5' TCTCAATGCTTCTTGTTCCG 3'<br>rv 5' CTAGATCCAATGCTGCCAGA 3'
MYB51	fw 5' CGAGGTTTCTTCACGAGGAT 3'<br>rv 5' CGTGGGATTCATCGATTATG 3'
