# Supplementary figures and images for: In roots of Arabidopsis thaliana, the damage-associated molecular pattern AtPep1 is a stronger elicitor of immune signalling than flg22 or the chitin heptamer
Source: PLoS One. 2017 Oct 3;12(10):e0185808. doi: 10.1371/journal.pone.0185808 (PMC5626561; doi:10.1371/journal.pone.0185808)

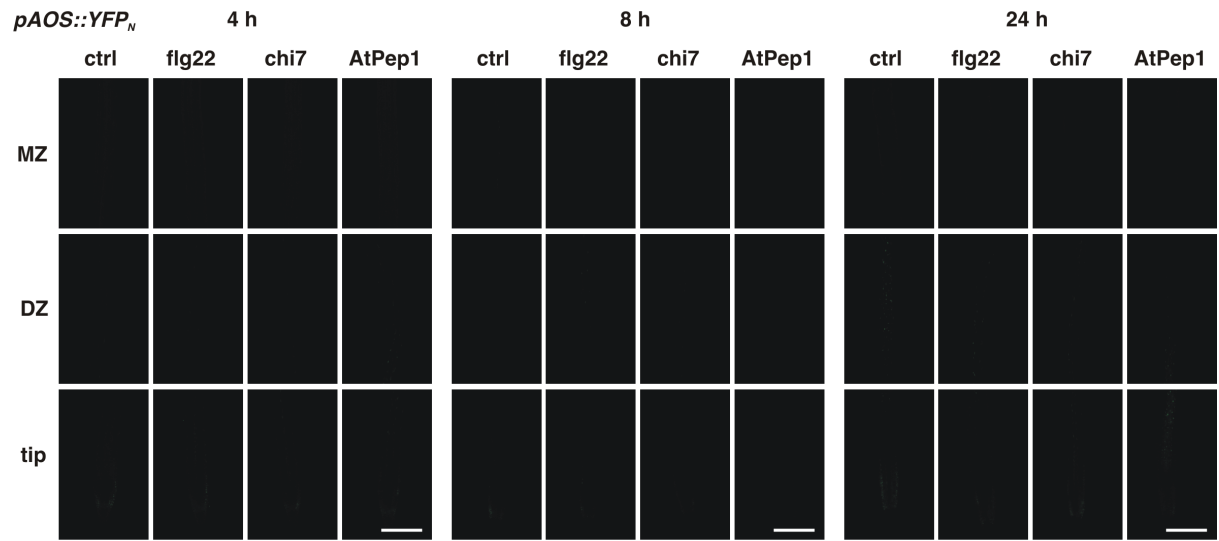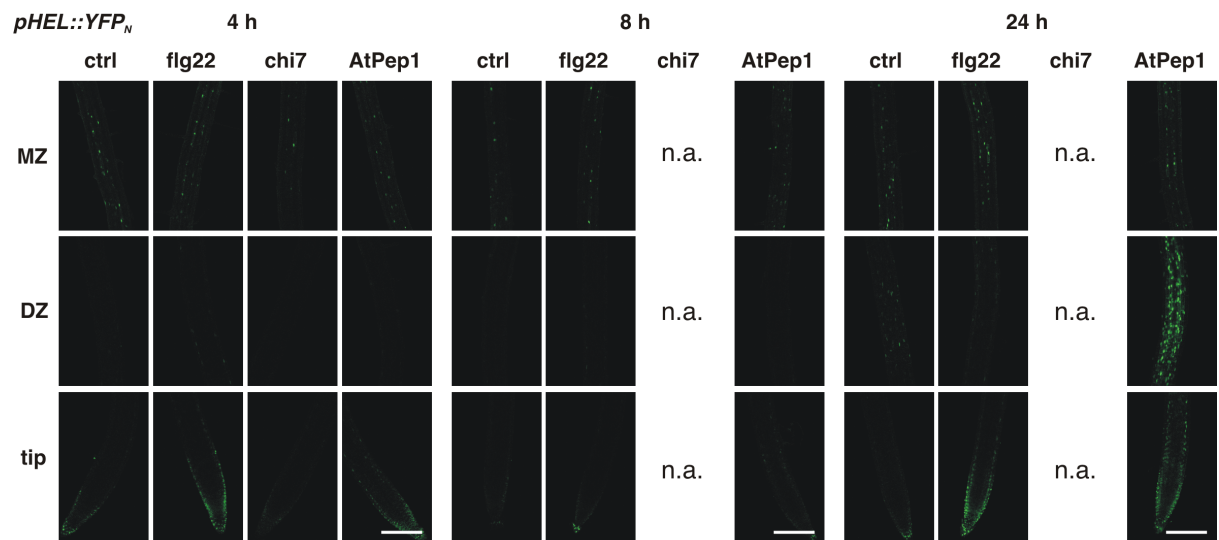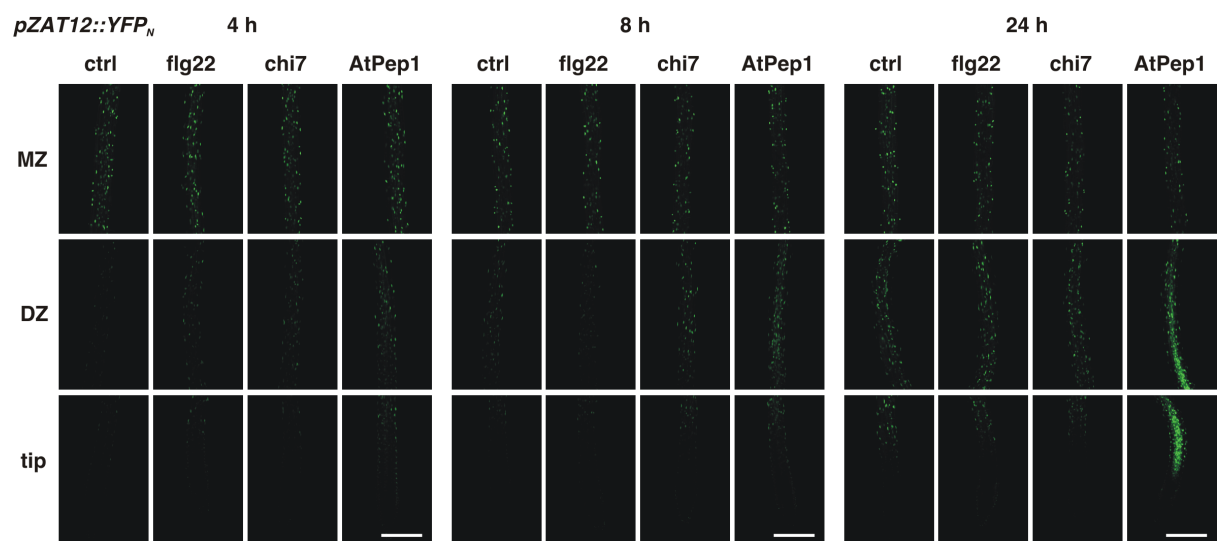

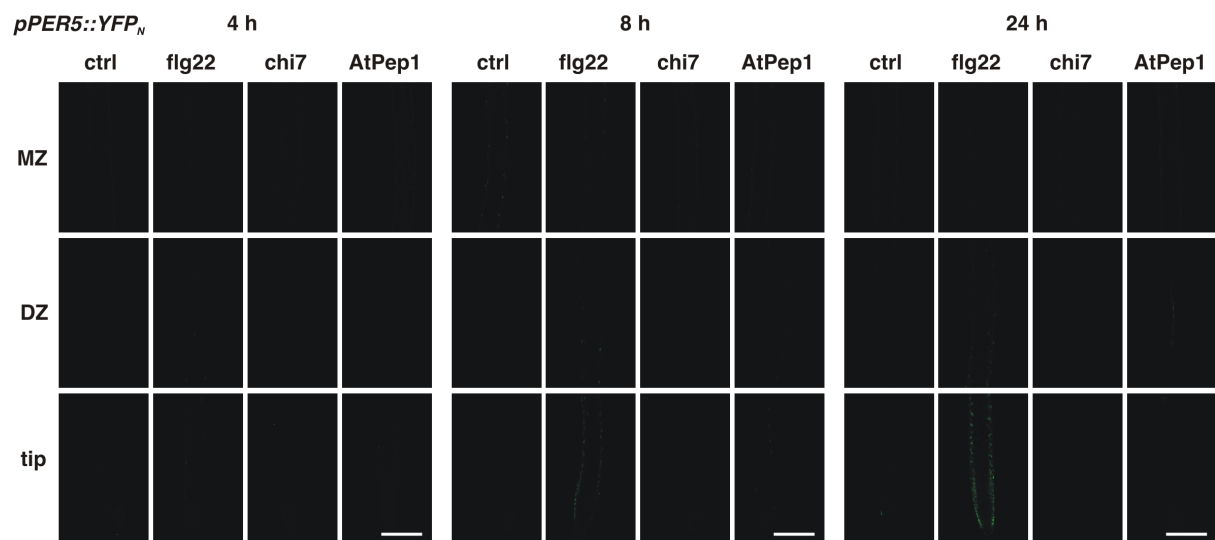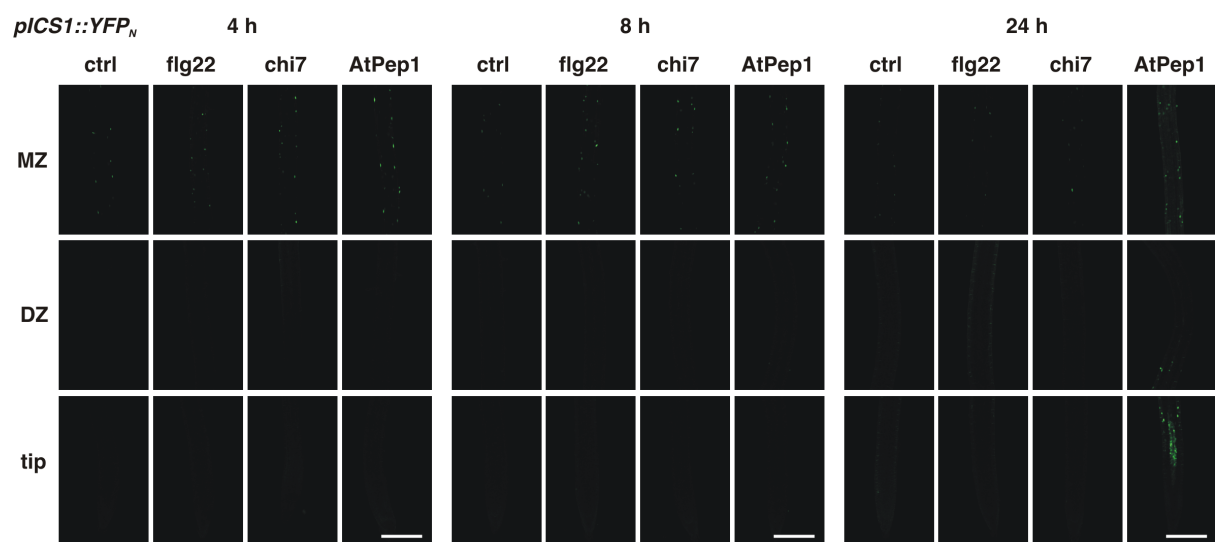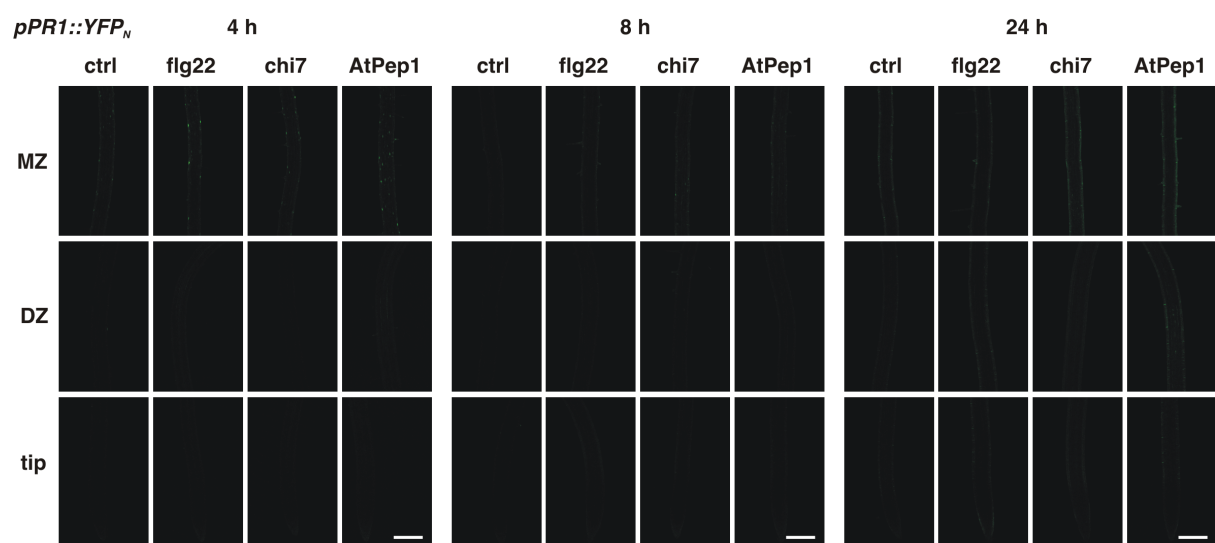

Supplement: S1 Fig — Roots were analysed following treatment with 100 nM flg22, chi7, AtPep1 or 0.5x MS as control. Scale bar 200 μm. (PDF) [file pone.0185808.s002.pdf]
