# Supplementary material for: In roots of Arabidopsis thaliana, the damage-associated molecular pattern AtPep1 is a stronger elicitor of immune signalling than flg22 or the chitin heptamer
Source: PLoS One. 2017 Oct 3;12(10):e0185808. doi: 10.1371/journal.pone.0185808 (PMC5626561; doi:10.1371/journal.pone.0185808)
Supplement: S2 Fig — Allele distribution of pepr1 and pepr2 in a segregating F2 population of a pepr1 pepr2 cross with promoter::YFPN lines selected for loss of root inhibition on 0.5x MS containing 100 nM AtPep1. Numbers represent individual plants genotyped for the respective pepr allele. (PDF) [file pone.0185808.s003.pdf]

S2 Fig. AtPEPR2 determines root growth inhibition by AtPep1.

Allele distribution of *pepr1* and *pepr2* in a segregating F2 population of a *pepr1 pepr2* cross with *promoter::YFP*$_N$ lines selected for loss of root inhibition on 0.5x MS containing 100 nM AtPep1. Numbers represent individual plants genotyped by PCR for the respective *pepr* T-DNA insertion.

	pepr1	*pepr2*
WT	14	-
heterozygous	47	6
homozygous	38	93
