# Supplementary material for: In roots of Arabidopsis thaliana, the damage-associated molecular pattern AtPep1 is a stronger elicitor of immune signalling than flg22 or the chitin heptamer
Source: PLoS One. 2017 Oct 3;12(10):e0185808. doi: 10.1371/journal.pone.0185808 (PMC5626561; doi:10.1371/journal.pone.0185808)
Supplement: S3 Fig — Roots were analysed following treatment with 100 nM flg22, chi7, AtPep1 or 0.5x MS as control. Scale bar 100 μm. Signal amplification might differ between developmental zones (S5 Fig). (PDF) [file pone.0185808.s004.pdf]

S3 Fig. Effects of elicitors on the expression of *promoter::YFP$_N$* in the root.
Roots were analysed following treatment with 100 nM flg22, chi7, AtPep1 or 0.5x MS as control. Scale bar 100 µm. Signal amplification might differ between developmental zones (S5 Fig).

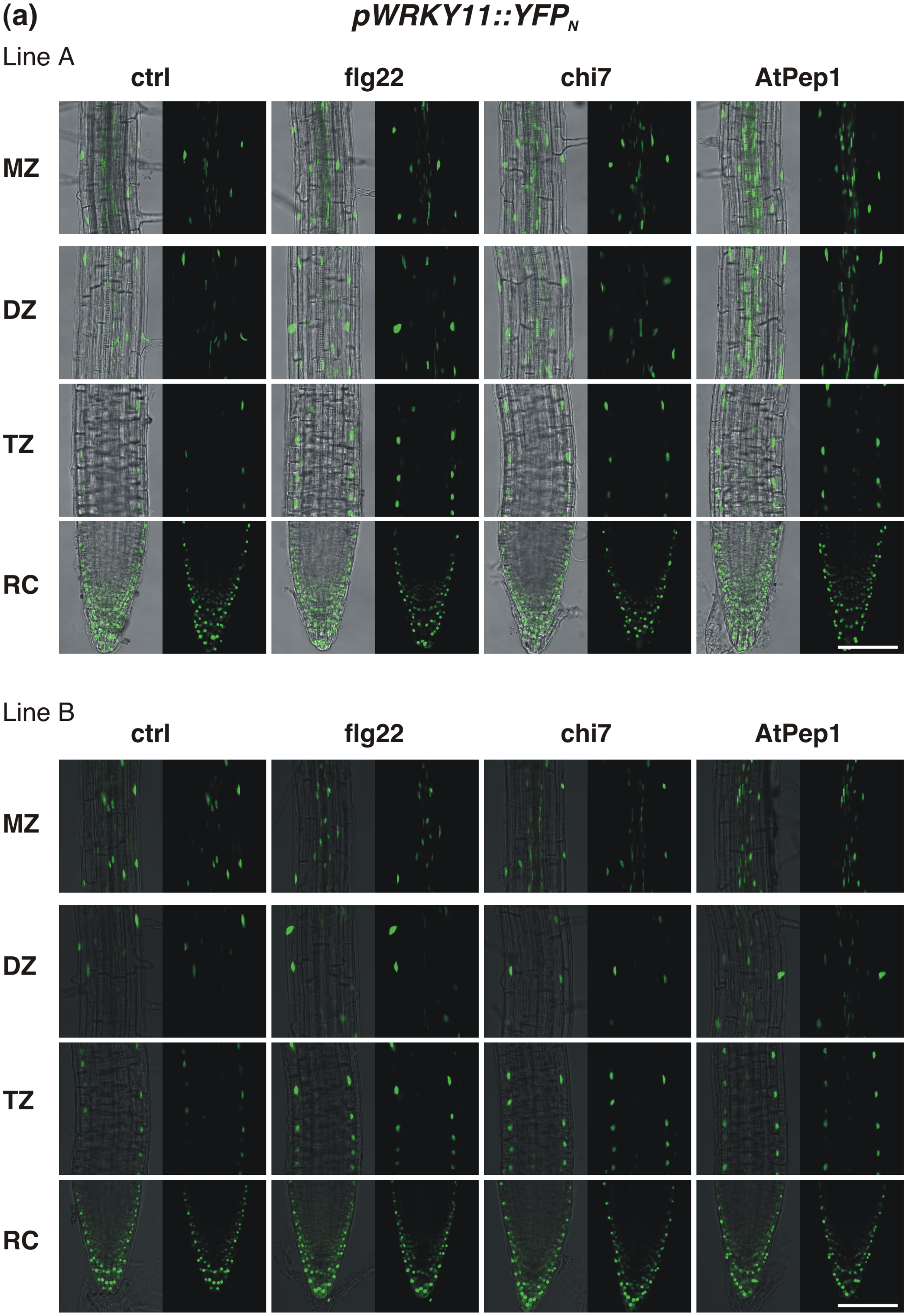

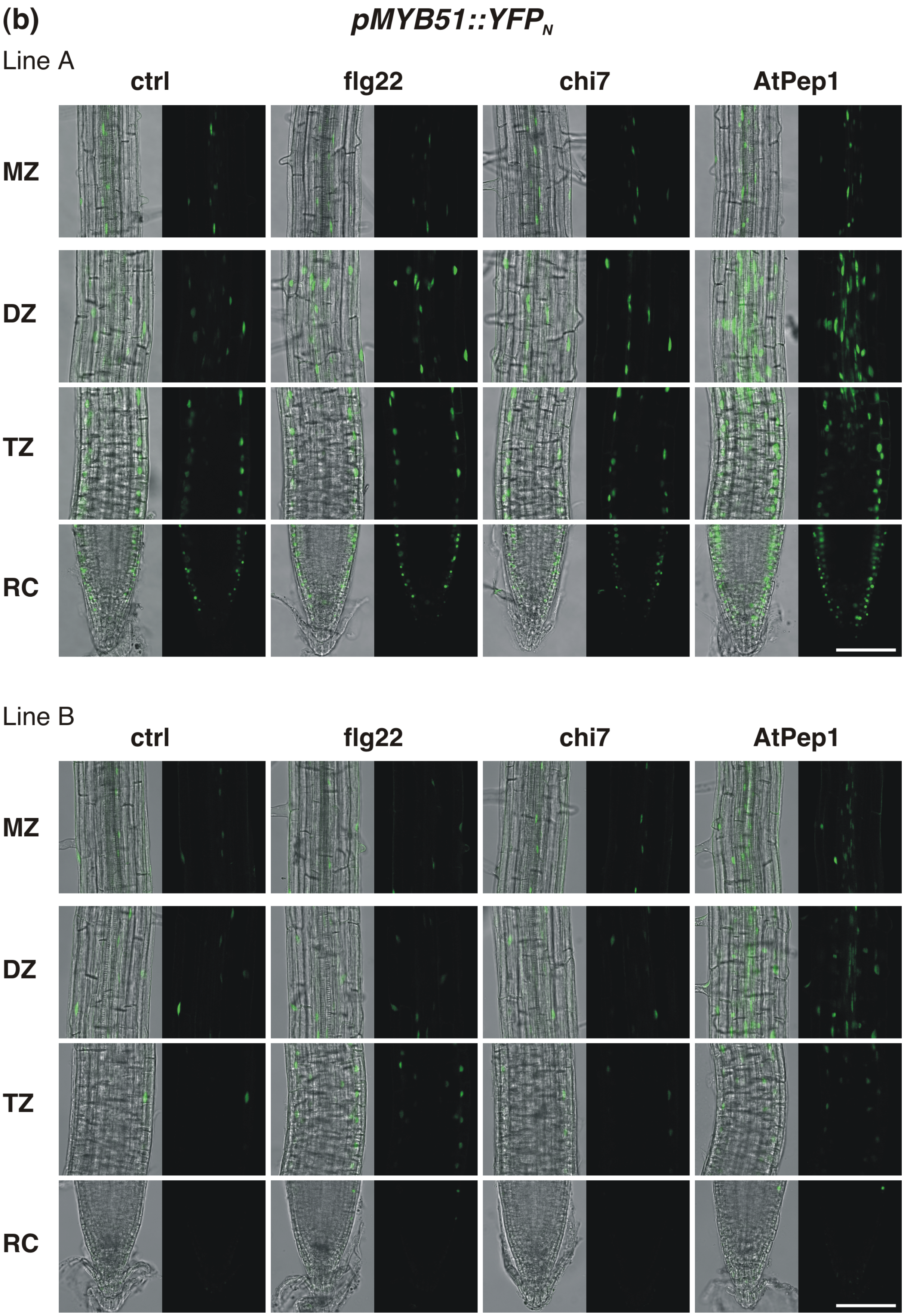
(b)
pMYB51::YFP$_N$
Line A
ctrl
flg22
chi7
AtPep1
MZ
DZ
TZ
RC
Line B
ctrl
flg22
chi7
AtPep1
MZ
DZ
TZ
RC

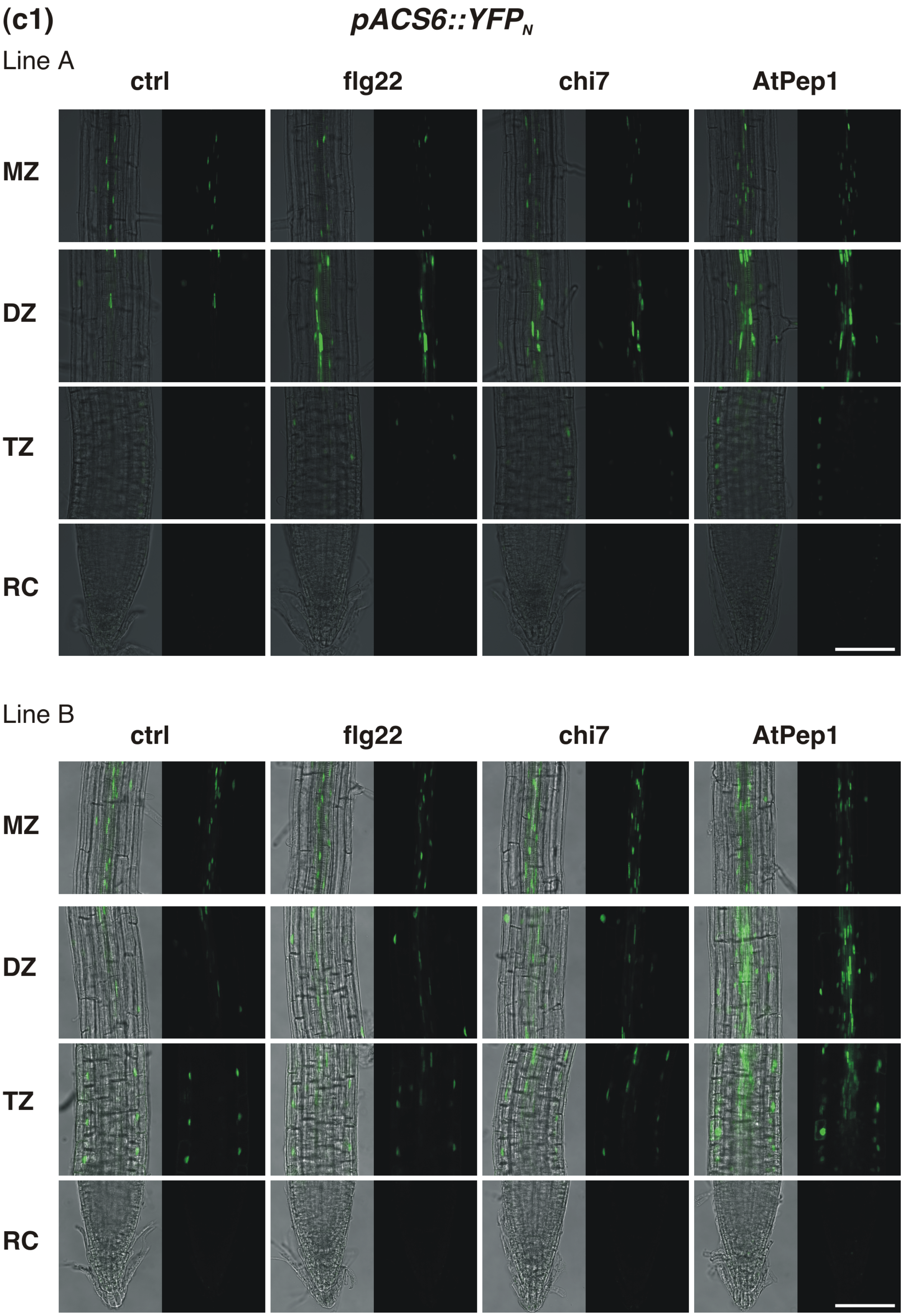

(c1)
pACS6::YFP_N
Line A
ctrl
flg22
chi7
AtPep1
MZ
DZ
TZ
RC
Line B
ctrl
flg22
chi7
AtPep1
MZ
DZ
TZ
RC

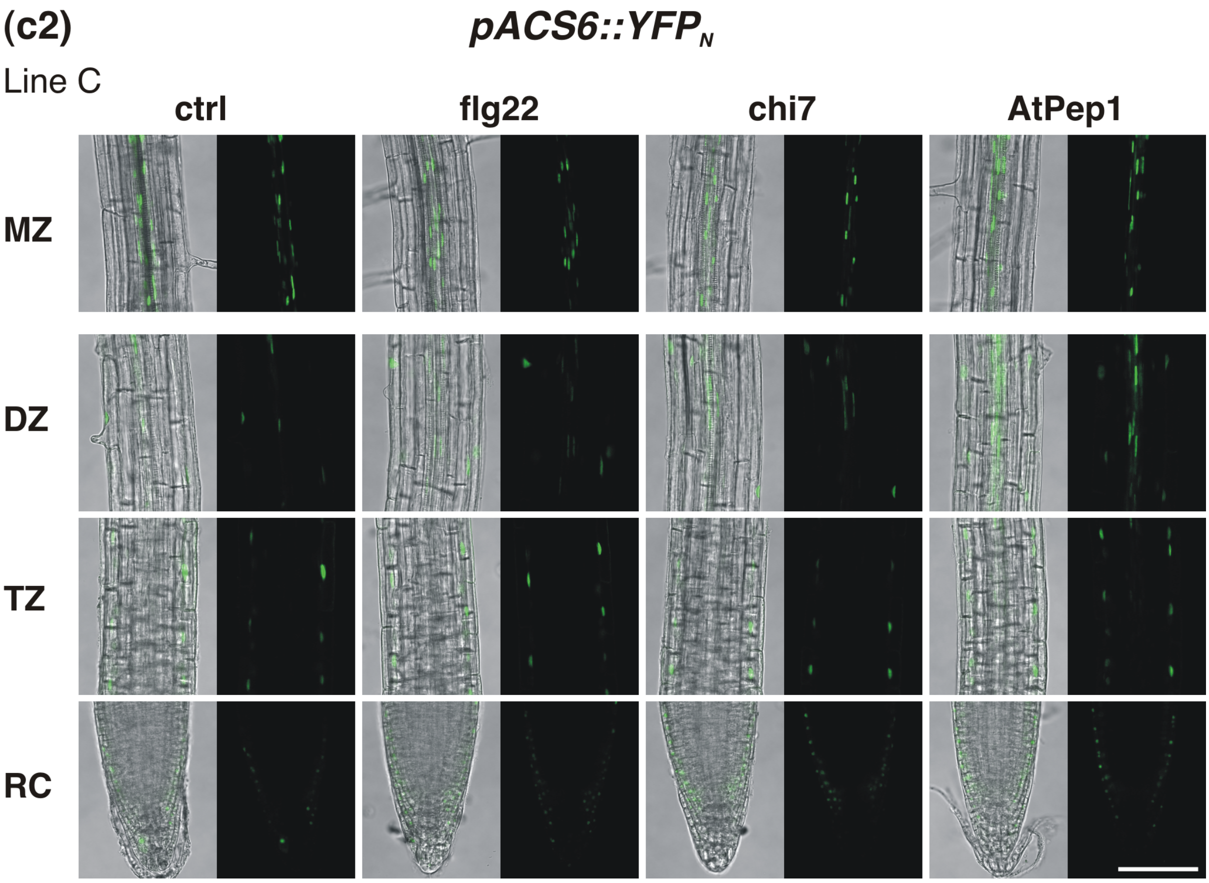
(c2)
pACS6::YFPN
Line C
ctrl
flg22
chi7
AtPep1
MZ
DZ
TZ
RC

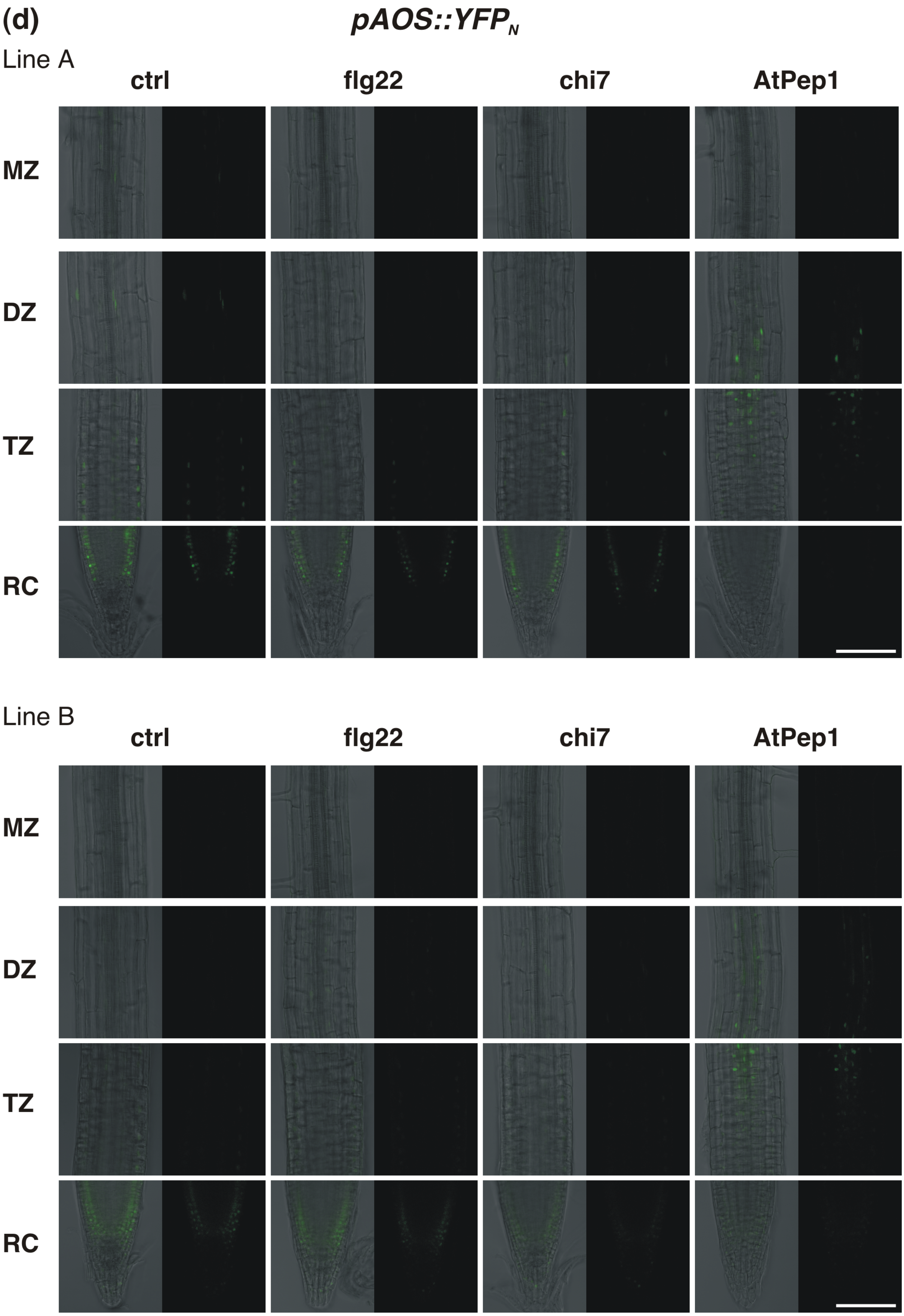
(d)
pAOS::YFP$_N$
Line A
ctrl
flg22
chi7
AtPep1
MZ
DZ
TZ
RC
Line B
ctrl
flg22
chi7
AtPep1
MZ
DZ
TZ
RC

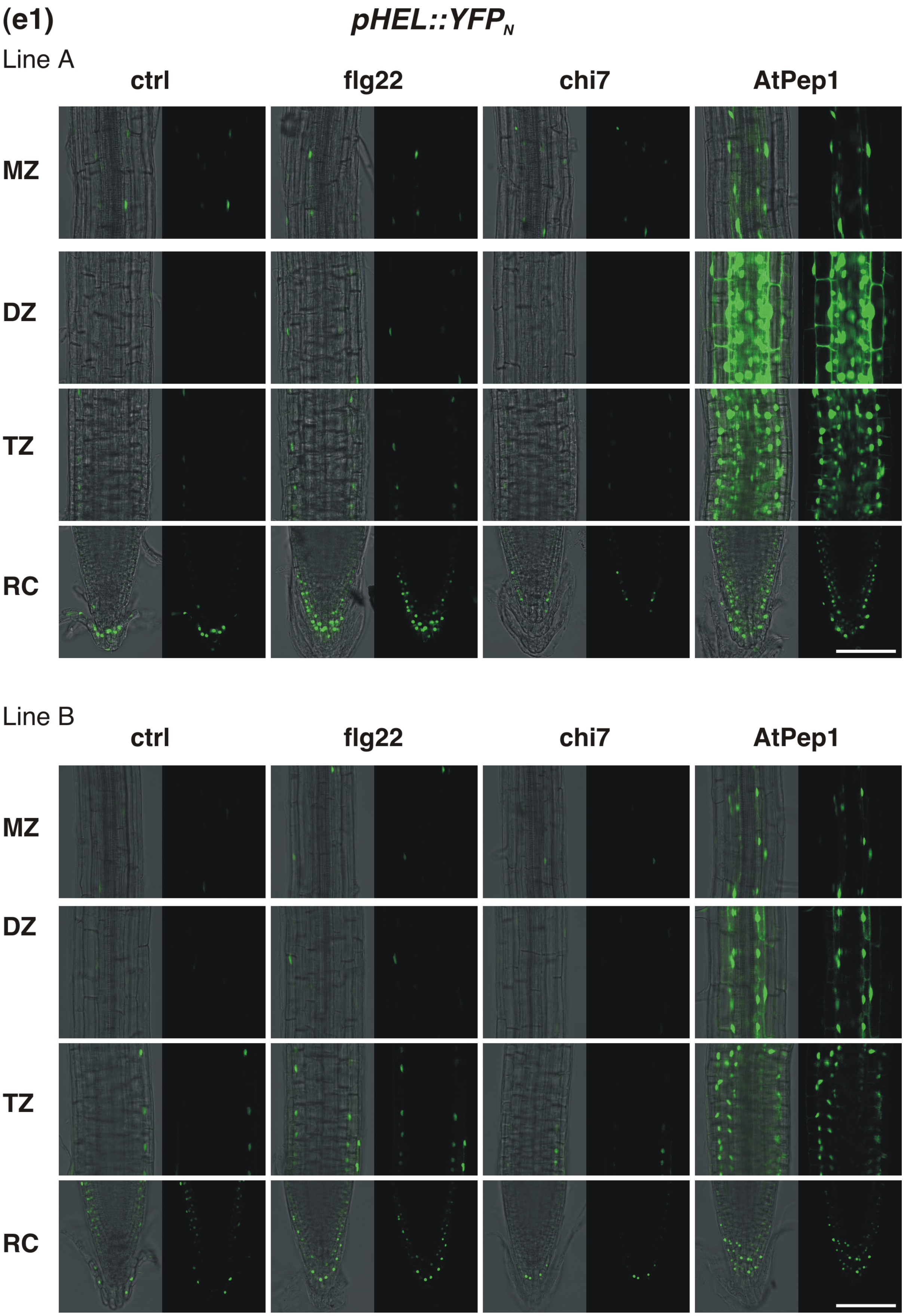
(e1)
pHEL::YFP$_N$
Line A
ctrl
flg22
chi7
AtPep1
MZ
DZ
TZ
RC
Line B
ctrl
flg22
chi7
AtPep1
MZ
DZ
TZ
RC

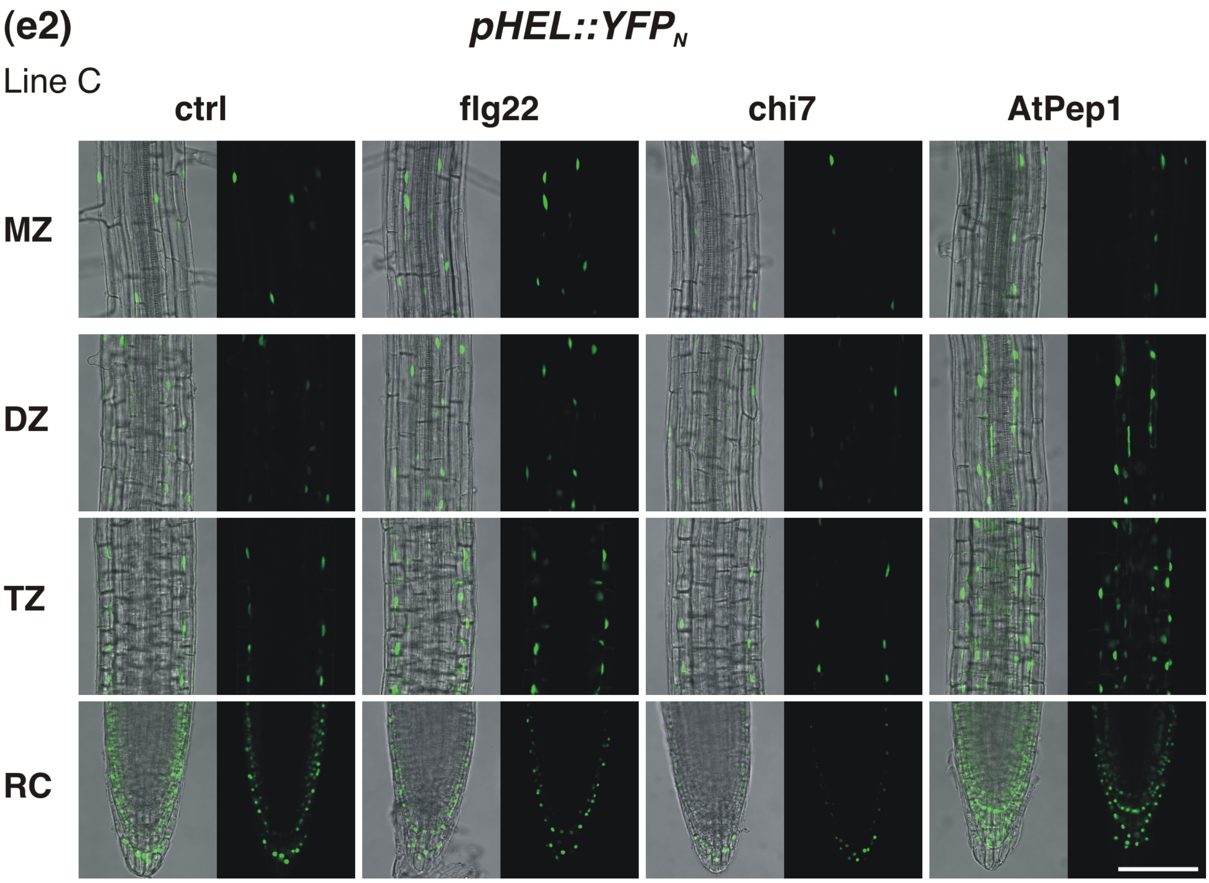
(e2)
pHEL::YFP_N
Line C
ctrl
flg22
chi7
AtPep1
MZ
DZ
TZ
RC

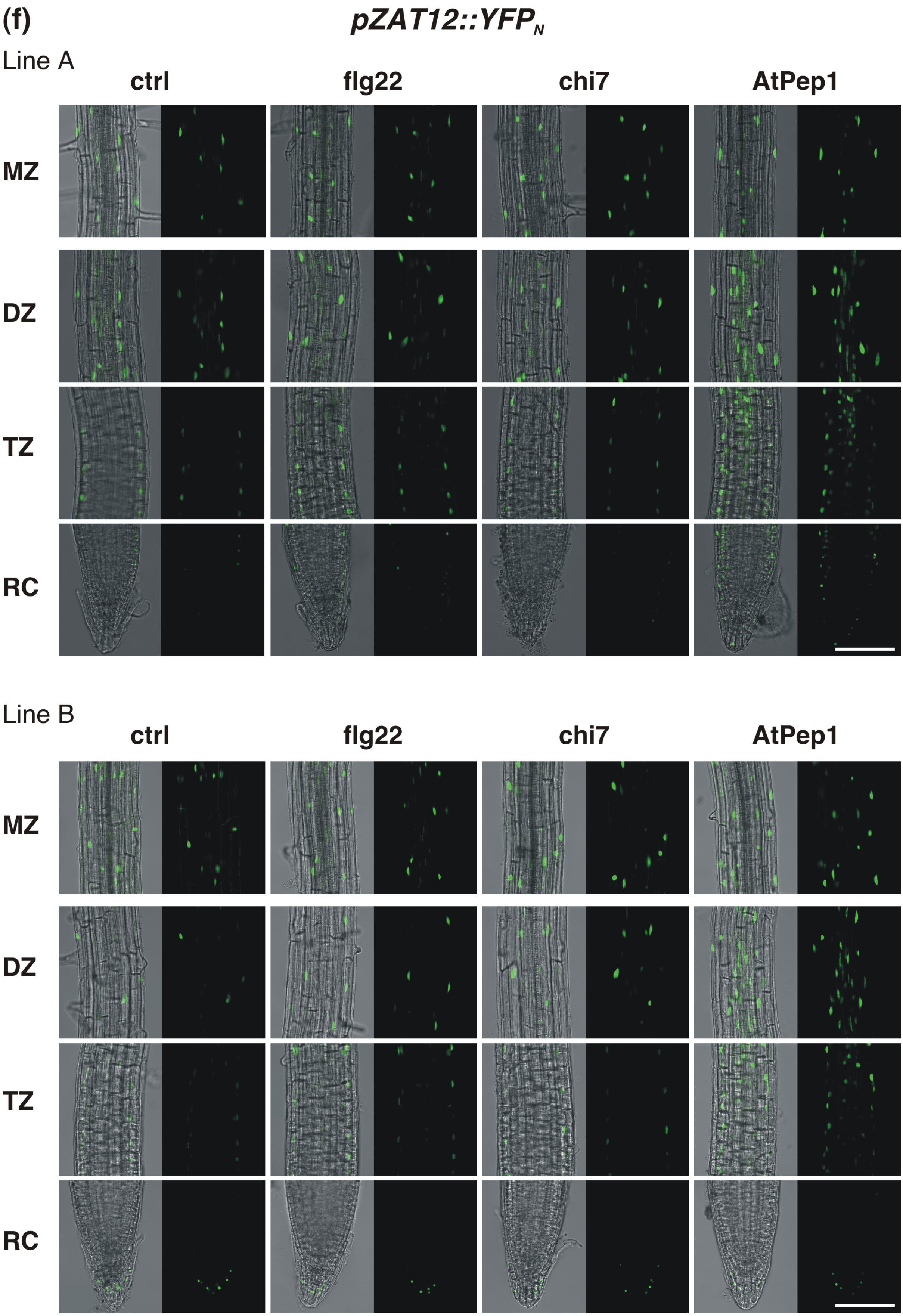

(f)
pZAT12::YFPN
Line A
ctrl
flg22
chi7
AtPep1
MZ
DZ
TZ
RC
Line B
ctrl
flg22
chi7
AtPep1
MZ
DZ
TZ
RC

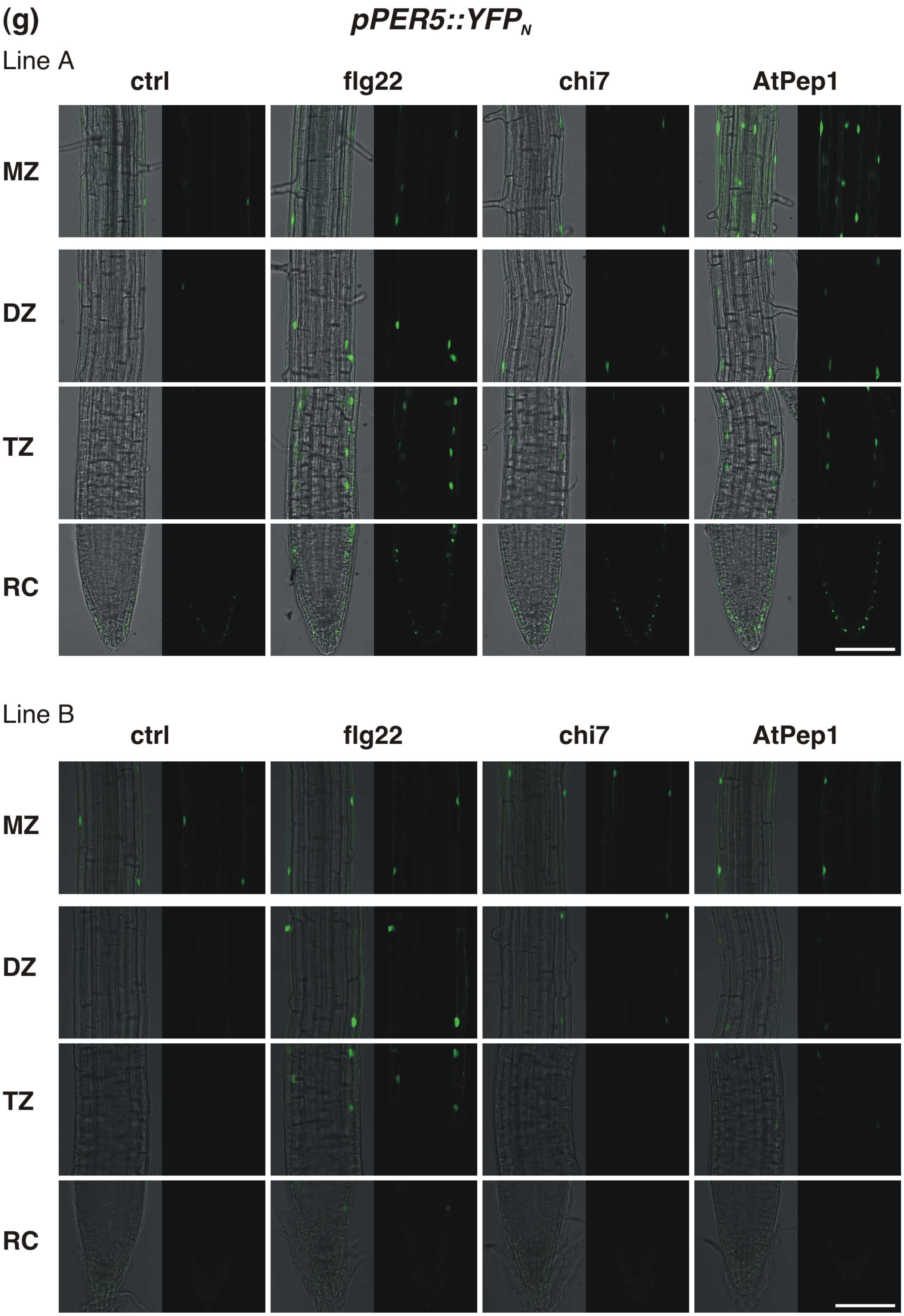
(g)
pPER5::YFP$_N$
Line A
ctrl
flg22
chi7
AtPep1
MZ
DZ
TZ
RC
Line B
ctrl
flg22
chi7
AtPep1
MZ
DZ
TZ
RC

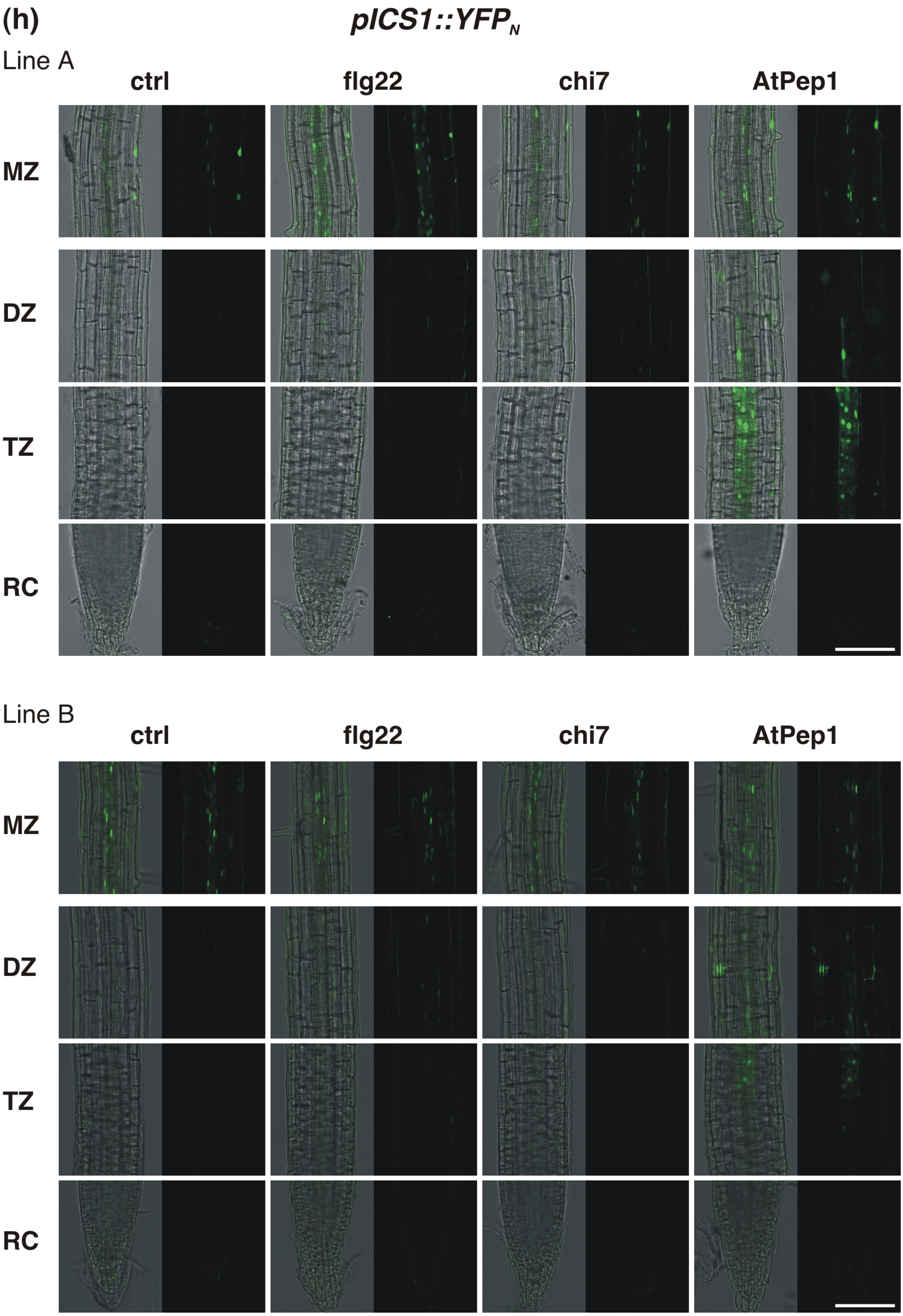

(h)
pICS1::YFP$_N$
Line A
ctrl
flg22
chi7
AtPep1
MZ
DZ
TZ
RC
Line B
ctrl
flg22
chi7
AtPep1
MZ
DZ
TZ
RC

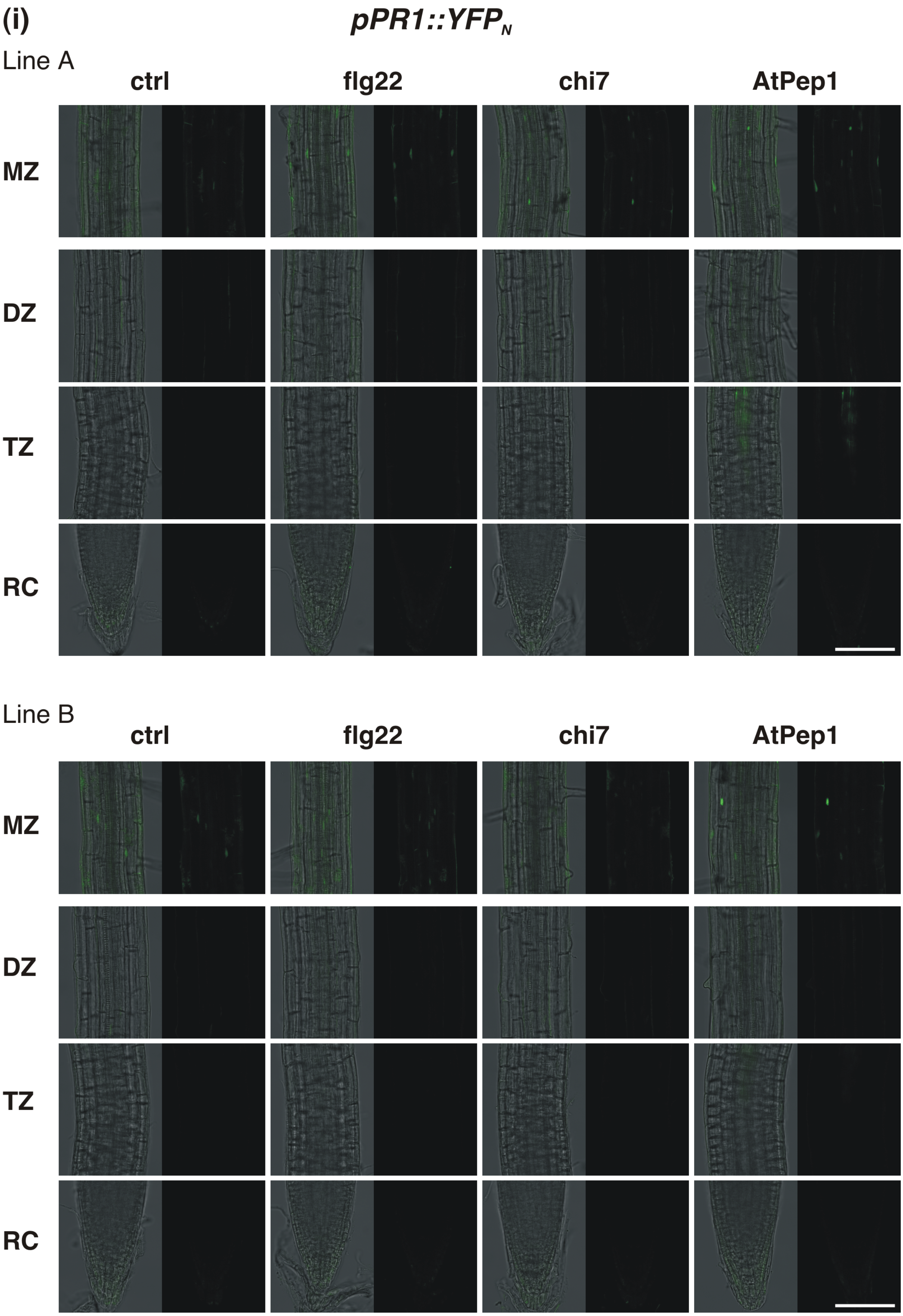
(i)
pPR1::YFPN
Line A
ctrl
flg22
chi7
AtPep1
MZ
DZ
TZ
RC
Line B
ctrl
flg22
chi7
AtPep1
MZ
DZ
TZ
RC
