# Supplementary material for: In roots of Arabidopsis thaliana, the damage-associated molecular pattern AtPep1 is a stronger elicitor of immune signalling than flg22 or the chitin heptamer
Source: PLoS One. 2017 Oct 3;12(10):e0185808. doi: 10.1371/journal.pone.0185808 (PMC5626561; doi:10.1371/journal.pone.0185808)

S4 Fig. Quantification of elicitor-triggered responses of defence-associated promoters in the root. Quantification of microscopic analysis of *promoter::YFP*$_N$ constructs in two independent lines of 7-days old seedlings following treatment with 100 nM flg22, chi7, AtPep1 or 0.5x MS as control. Images were analysed using Fiji. Bars represent the mean of ≥ 3 images ± SD. An asterisk indicates a mean of two values.

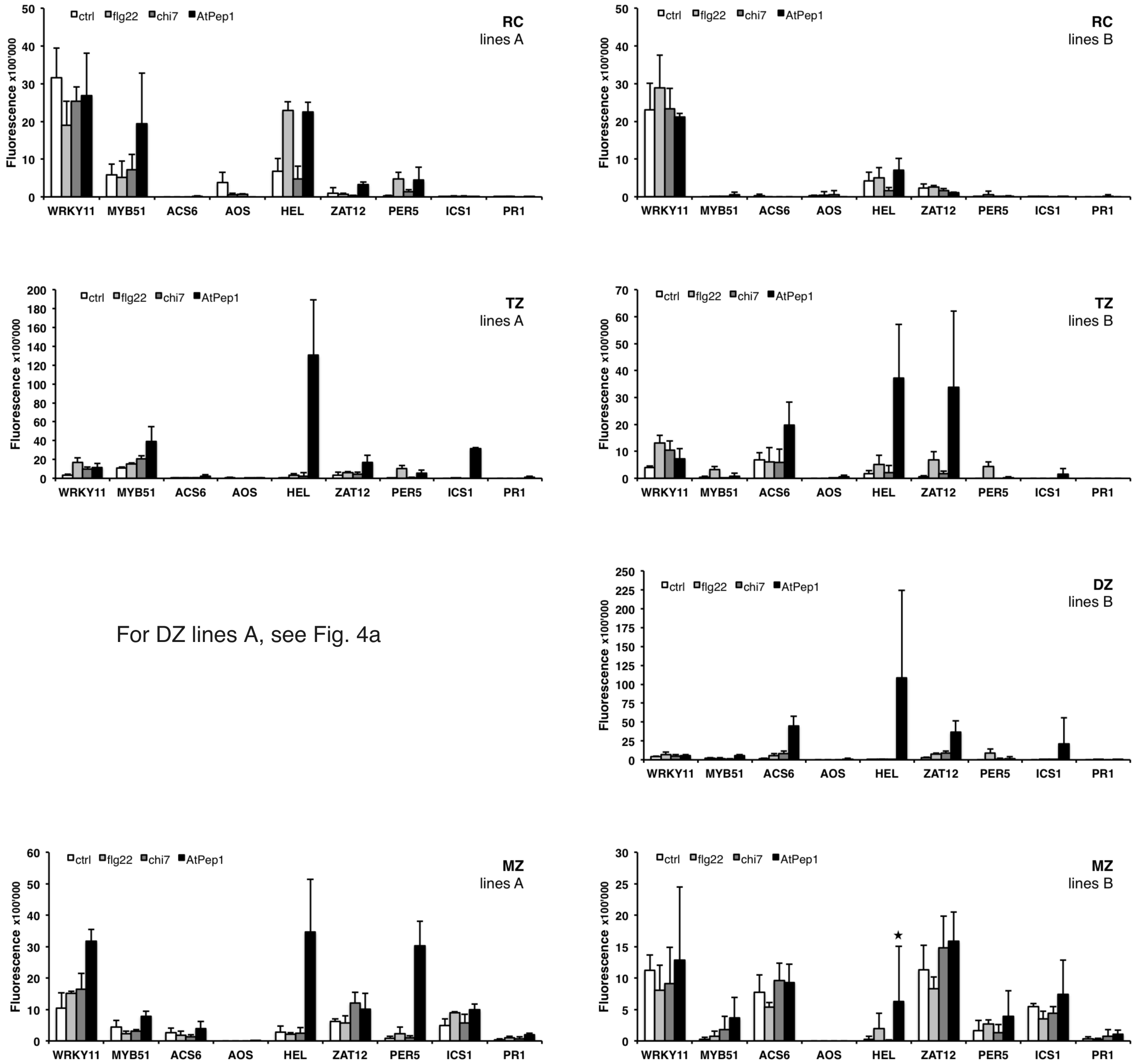

Supplement: S4 Fig — Quantification of microscopic analysis of promoter::YFPN constructs in two independent lines of 7-days old seedlings following treatment with 100 nM flg22, chi7, AtPep1 or 0.5x MS as control. Images were analysed using Fiji. Bars represent the mean of ≥ 3 images ± SD. An asterisk indicates a mean of two values. (PDF) [file pone.0185808.s005.pdf]
