# Supplementary material for: In roots of Arabidopsis thaliana, the damage-associated molecular pattern AtPep1 is a stronger elicitor of immune signalling than flg22 or the chitin heptamer
Source: PLoS One. 2017 Oct 3;12(10):e0185808. doi: 10.1371/journal.pone.0185808 (PMC5626561; doi:10.1371/journal.pone.0185808)
Supplement: S5 Fig — (a) Overview of inductive and suppressive responses of promoter::YFPN constructs expressed in 7-days old seedlings following treatment with 100 nM flg22, chi7, AtPep1 or 0.5x MS as control. Numbers represent the factors calculated as fold-change for 2 independent transformation lines per construct. A third line was analysed for ACS6::YFPN and HEL::YFPN constructs to confirm whether or not chi7 can cause a suppression of these markers. Black colour of a field indicates induction, white colour suppression and grey colour non-significant changes. (b) Table summarizing the amplification settings (gain) used for confocal microscopic analysis of promoter::YFPN constructs listed as an indicator for the expression level of the respective construct. (PDF) [file pone.0185808.s006.pdf]

S5 Fig. Summary of elicitor-triggered responses and amplification settings used for microscopic analysis. (a) Overview of inductive and suppressive responses of *promoter::YFP*$_N$ constructs expressed in 7-days old seedlings following treatment with 100 nM flg22, chi7, AtPep1 or 0.5x MS as control. Numbers represent the factors calculated as fold-change for 2 independent transformation lines per construct. A third line was analysed for *ACS6::YFP*$_N$ and *HEL::YFP*$_N$ constructs to confirm whether or not chi7 can cause a suppression of these markers. Black colour of a field indicates induction, white colour suppression and grey colour non-significant changes. (b**)** Table summarizing the amplification settings (gain) used for confocal microscopic analysis of *promoter::YFP*$_N$ constructs listed as an indicator for the expression level of the respective construct.

(a)

■ induction > 1.5 □ suppression < 0.67 ▩ no change

fold-change	flg22				chi7				Pep1			
	RC	TZ	DZ	MZ	RC	TZ	DZ	MZ	RC	TZ	DZ	MZ
WRKY11	0.60/ 1.26	**4.72/ 3.30**	**2.03/ 1.83**	1.46/ 0.72	0.80/ 1.01	**2.86/ 2.64**	1.34/ 1.25	1.59/ 0.81	0.85/ 0.92	**3.27/ 1.84**	3.73/ 1.47	3.06/ 1.14
MYB51	0.88/ 1.00/	1.38/ 6.44/	1.72/ 0.72/	0.50/ 1.47/	1.24/ 1.00/	1.86/ 1.00/	1.53/ 0.35/	0.69/ 3.61/	3.34/ 1.20/	**3.54/ 1.68/**	**6.31/ 2.19/**	**1.77/ 7.30/**
ACS6	1.00/ 1.00/ 1.24	1.00/ 0.88/ 1.98	**10.83/ 4.70/ 1.19**	0.67/ 0.70/ 0.71	1.00/ 1.00/ 1.90	1.00/ 0.86/ 1.52	**6.02/ 7.35/ 1.97**	**0.48/ 1.24/ 0.59**	1.00/ 1.00/ 1.89	**4.61/ 2.86/ 2.01**	**29.41/ 39.08/ 9.19**	1.50/ 1.21/ 1.10
AOS	0.13/ 1.00	1.00/ 1.00	1.00/ 1.00	1.00/ 1.00	0.17/ 1.25	1.00/ 1.00	1.00/ 1.00	1.00/ 1.00	0.13/ 1.00	1.00/ 1.24	**4.30/ 1.76**	1.00/ 1.00
HEL	3.39/ 1.18/ 0.57	**7.04/ 2.94/ 1.98**	**7.56/ 1.00/ 2.05**	0.73/ 3.92/ 1.29	**0.70/ 0.37/ 0.24**	4.38/ 1.21/ 0.95	1.00/ 1.00/ 0.39	0.85/ 1.00/ 0.42	**3.34/ 1.66/ 1.09**	**260.97/ 21.58/ 4.91**	**764.64/ 216.70/ 6.79**	**12.14/ 12.48/ 1.44**
ZAT12	0.70/ 1.08	**1.76/ 12.59**	**1.89/ 2.50**	0.92/ 0.73	0.48/ 0.73	1.24/ 3.00	**1.51/ 2.98**	1.97/ 1.31	3.13/ 0.44	**4.88/ 61.52**	**5.33/ 12.60**	1.65/ 1.40
PER5	9.66/ 1.10	**21.14/ 8.85**	**21.34/ 17.35**	**2.73/ 1.62**	2.76/ 1.00	1.00/ 1.00	**2.10/ 1.76**	1.17/ 0.81	8.87/ 1.00	10.99/ 1.00	**11.08/ 3.08**	**35.35/ 2.34**
ICS1	1.00/ 1.00	1.00/ 1.00	1.00/ 1.00	1.83/ 0.65	1.00/ 1.00	1.00/ 1.00	1.00/ 1.00	1.17/ 0.81	1.00/ 1.00	**63.04/ 3.26**	**27.70/ 42.11**	2.03/ 1.37
PR1	1.00/ 1.00	1.00/ 1.00	1.00/ 1.00	1.94/ 1.00	1.00/ 1.00	1.00/ 1.00	1.00/ 1.00	1.24/ 1.55	1.00/ 1.00	2.03/ 1.00	1.00/ 1.00	**3.93/ 2.13**

(b)

Amplification	RC	TZ	DZ	MZ
WRKY11	700/ 800	750/ 800	800/ 800	800/ 800
MYB51	1000/ 1100	1100/ 1200	1100/ 1200	950/ 1200
ACS6	1000/ 950/ 950	1000/ 950/ 950	900/ 775/ 950	700/ 650/ 720
AOS	1000/ 1200	1000/ 1200	1100/ 1200	1100/ 1200
HEL	850/ 850/ 750	1100/ 950/ 900	1100/ 950/ 900	850/ 800/ 750
ZAT12	750/ 750	750/ 850	700/ 750	700/ 800
PER5	950/ 1100	950/ 1100	950/ 1100	1100/ 1100
ICS1	1200/ 1200	1200/ 1200	1200/ 1200	1200/ 1200
PR1	1200/ 1200	1200/ 1200	1200/ 1200	1200/ 1200
