# Supplementary material for: In roots of Arabidopsis thaliana, the damage-associated molecular pattern AtPep1 is a stronger elicitor of immune signalling than flg22 or the chitin heptamer
Source: PLoS One. 2017 Oct 3;12(10):e0185808. doi: 10.1371/journal.pone.0185808 (PMC5626561; doi:10.1371/journal.pone.0185808)

S6 Fig. Quantification of *promoter::*YFP_N constructs at the infection site of roots invaded by *F. oxysporum*.

(a) Quantification of microscopic analysis of *F. oxysporum* - infected roots expressing *promoter::*YFP_N constructs in independent transformation lines when compared to Fig. 6. Bars represent the mean of ≥ 6 images ± SE. (b) Quantification of microscopic analysis of *pHEL::*YFP_N, *pPER5::*YFP_N and *pICS1::*YFP_N constructs using Fiji. Bars represent the mean of ≥ 20 images ± SE. Statistical analysis was performed using a Student's t-test: * $p < 0.05$, ** $p < 0.01$, *** $p < 0.001$. (b.t.) indicates that all signals were below the autofluorescence threshold.

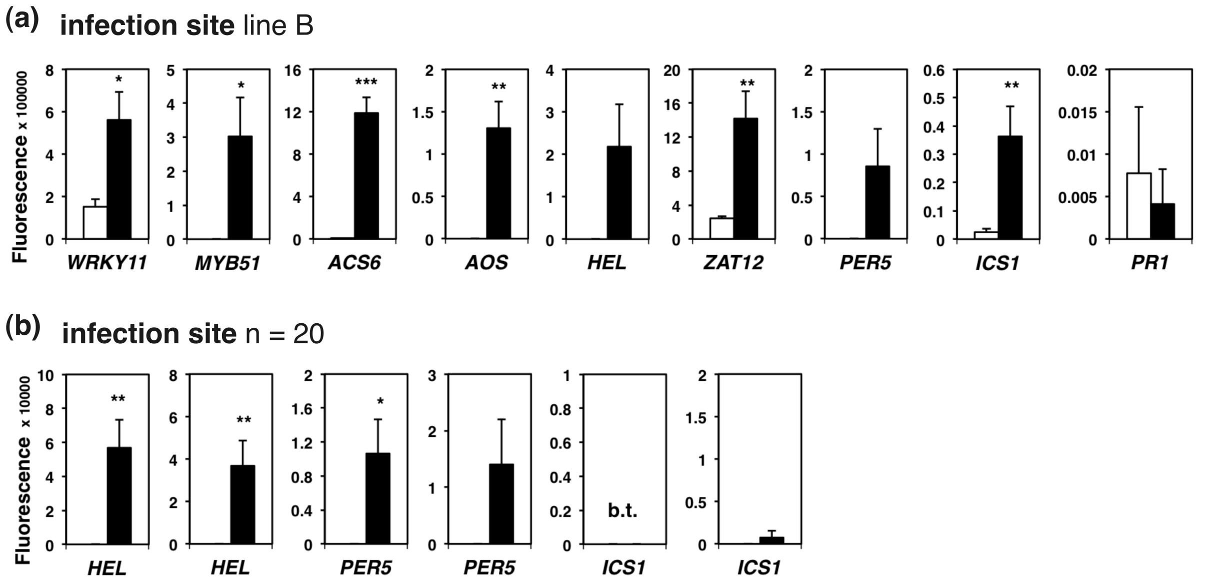

Supplement: S6 Fig — (a) Quantification of microscopic analysis of F. oxysporum—infected roots expressing promoter::YFPN constructs in independent transformation lines when compared to Fig 6. Bars represent the mean of ≥ 6 images ± SE. (b) Quantification of microscopic analysis of pHEL::YFPN, pPER5::YFPN and pICS1::YFPN constructs using Fiji. Bars represent the mean of ≥ 20 images ± SE. Statistical analysis was performed using a Student’s t-test: * p < 0.05, ** p < 0.01, *** p < 0.001. (b.t.) indicates that all signals were below the autofluorescence threshold. (PDF) [file pone.0185808.s007.pdf]
