# Supplementary material for: In roots of Arabidopsis thaliana, the damage-associated molecular pattern AtPep1 is a stronger elicitor of immune signalling than flg22 or the chitin heptamer
Source: PLoS One. 2017 Oct 3;12(10):e0185808. doi: 10.1371/journal.pone.0185808 (PMC5626561; doi:10.1371/journal.pone.0185808)

S7 Fig. Induction of *promoter::YFP$_N$* constructs adjacent to invading hyphae of GFP-labelled *F. oxysporum.*

Microscopic analysis of the responses of 12-day old roots in the mature part 2 days after inoculation with spores from *F. oxysporum*. Fluorescence derived from *promoter::YFP$_N$* constructs is localized in the root nuclei while fungal hyphae are green filamentous structures. Bar 100 µm.

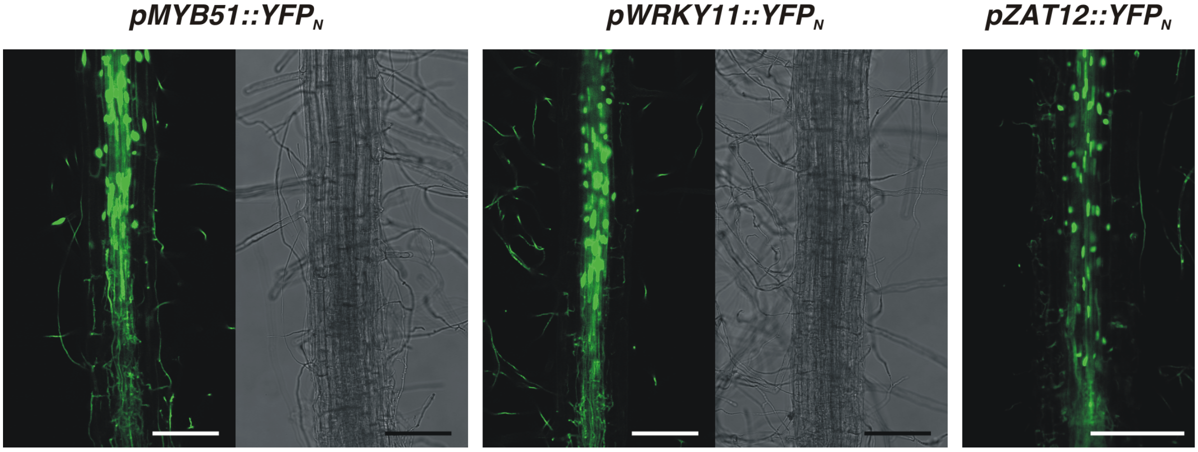

Supplement: S7 Fig — Microscopic analysis of the responses of 12-day old roots in the mature part 2 days after inoculation with spores from F. oxysporum. Fluorescence derived from promoter::YFPN constructs is localized in the root nuclei while fungal hyphae are green filamentous structures. Bar 100 μm. (PDF) [file pone.0185808.s008.pdf]
