# Supplementary material for: In roots of Arabidopsis thaliana, the damage-associated molecular pattern AtPep1 is a stronger elicitor of immune signalling than flg22 or the chitin heptamer
Source: PLoS One. 2017 Oct 3;12(10):e0185808. doi: 10.1371/journal.pone.0185808 (PMC5626561; doi:10.1371/journal.pone.0185808)

S8 Fig. Expression of *promoter::YFP*$_N$ constructs in the mature part of roots infected with *F. oxysporum*.

(a) Microscopic analysis of the responses of 12-day old roots in the mature part 2 days after inoculation with spores from *F. oxysporum*. Fluorescence derived from *promoter::YFP*$_N$ constructs is localized in the root nuclei and shown in green, while propidium iodide was used as a counterstain of cell walls and dead cells and is shown in red. Bar 100 µm. (b) Quantification of microscopic analysis of *promoter::YFP*$_N$ constructs as depicted in (A) using Fiji. Bars represent the mean of ≥ 6 images ± SE. (C) Quantification of microscopic analysis of *F. oxysporum* - infected roots expressing *promoter::YFP*$_N$ constructs in independent transformation lines when compared to Fig. 6A+B. Bars represent the mean of ≥ 6 images ± SE. Statistical analysis was performed using a Student's t-test: * $p < 0.05$, ** $p < 0.01$. (b.t.) indicates that all signals were below the autofluorescence threshold.

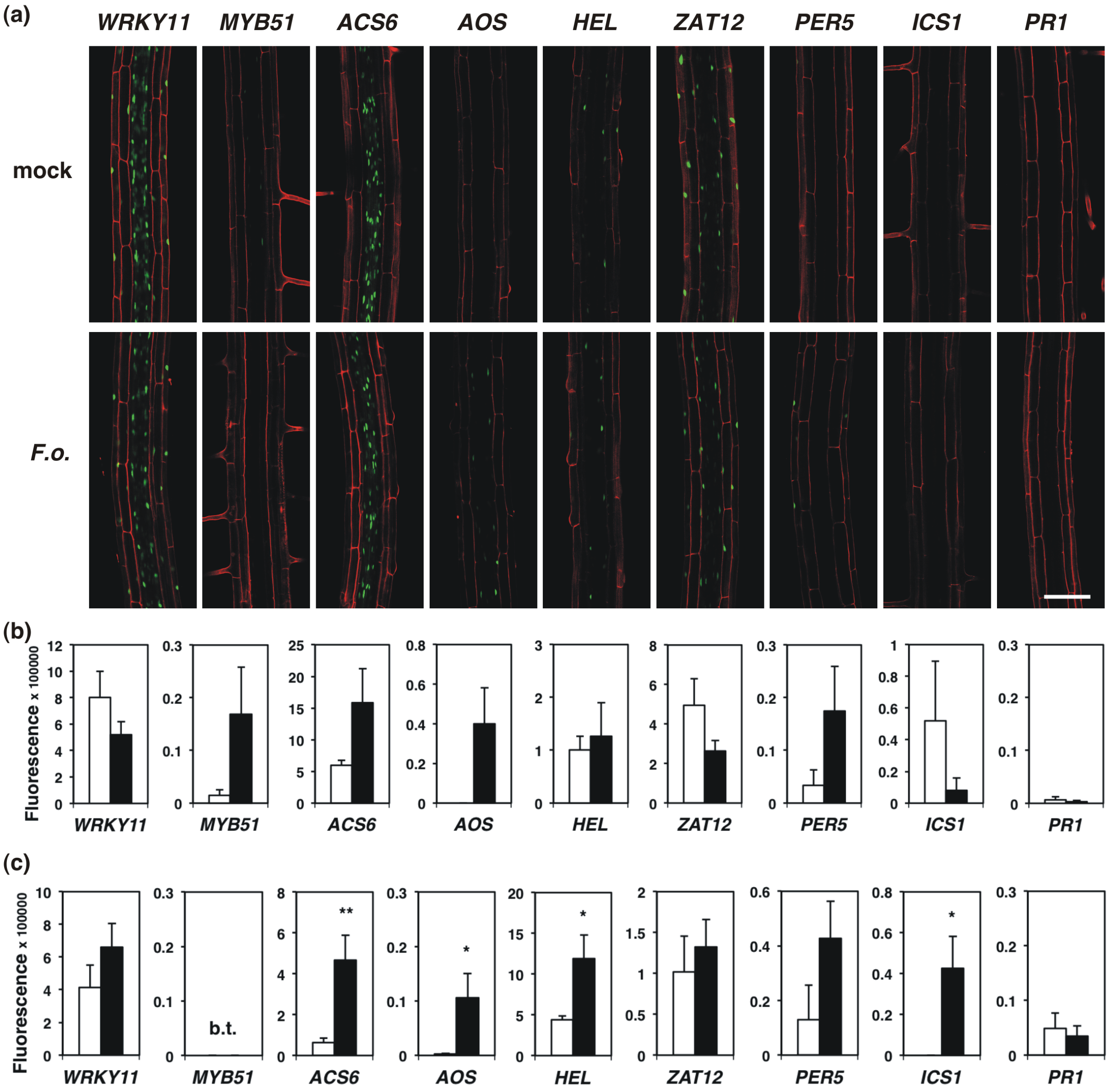

Supplement: S8 Fig — (a) Microscopic analysis of the responses of 12-day old roots in the mature part 2 days after inoculation with spores from F. oxysporum. Fluorescence derived from promoter::YFPN constructs is localized in the root nuclei and shown in green, while propidium iodide was used as a counterstain of cell walls and dead cells and is shown in red. Bar 100 μm. (b) Quantification of microscopic analysis of promoter::YFPN constructs as depicted in (A) using Fiji. Bars represent the mean of ≥ 6 images ± SE. (C) Quantification of microscopic analysis of F. oxysporum—infected roots expressing promoter::YFPN constructs in independent transformation lines when compared to Fig 6A and 6B. Bars represent the mean of ≥ 6 images ± SE. Statistical analysis was performed using a Student’s t-test: * p < 0.05, ** p < 0.01. (b.t.) indicates that all signals were below the autofluorescence threshold. (PDF) [file pone.0185808.s009.pdf]
