# Supplementary material for: In roots of Arabidopsis thaliana, the damage-associated molecular pattern AtPep1 is a stronger elicitor of immune signalling than flg22 or the chitin heptamer
Source: PLoS One. 2017 Oct 3;12(10):e0185808. doi: 10.1371/journal.pone.0185808 (PMC5626561; doi:10.1371/journal.pone.0185808)

S9 Fig. Expression of MAMP/DAMP receptor genes in roots.
Data were obtained from a public database (Genevestigator).

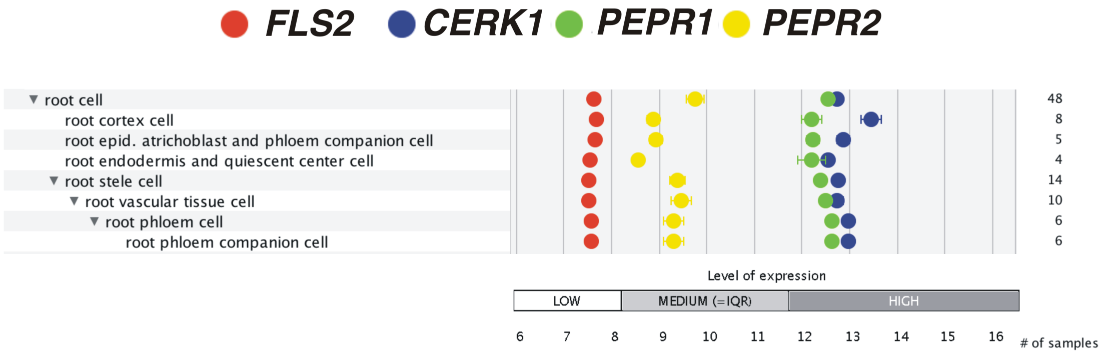

Supplement: S9 Fig — (PDF) [file pone.0185808.s010.pdf]
